# Topographic Evolution and Climate Aridification during Continental Collision: Insights from Computer Simulations

**DOI:** 10.1371/journal.pone.0132252

**Published:** 2015-08-05

**Authors:** Daniel Garcia-Castellanos, Ivone Jiménez-Munt

**Affiliations:** Group of Dynamics of the Lithosphere, Instituto de Ciencias de la Tierra Jaume Almera (ICTJA-CSIC), Barcelona, Spain; ETH, SWITZERLAND

## Abstract

How do the feedbacks between tectonics, sediment transport and climate work to shape the topographic evolution of the Earth? This question has been widely addressed via numerical models constrained with thermochronological and geomorphological data at scales ranging from local to orogenic. Here we present a novel numerical model that aims at reproducing the interaction between these processes at the continental scale. For this purpose, we combine in a single computer program: 1) a thin-sheet viscous model of continental deformation; 2) a stream-power surface-transport approach; 3) flexural isostasy allowing for the formation of large sedimentary foreland basins; and 4) an orographic precipitation model that reproduces basic climatic effects such as continentality and rain shadow. We quantify the feedbacks between these processes in a synthetic scenario inspired by the India-Asia collision and the growth of the Tibetan Plateau. We identify a feedback between erosion and crustal thickening leading locally to a <50% increase in deformation rates in places where orographic precipitation is concentrated. This climatically-enhanced deformation takes place preferentially at the upwind flank of the growing plateau, specially at the corners of the indenter (syntaxes). We hypothesize that this may provide clues for better understanding the mechanisms underlying the intriguing tectonic aneurisms documented in the Himalayas. At the continental scale, however, the overall distribution of topographic basins and ranges seems insensitive to climatic factors, despite these do have important, sometimes counterintuitive effects on the amount of sediments trapped within the continent. The dry climatic conditions that naturally develop in the interior of the continent, for example, trigger large intra-continental sediment trapping at basins similar to the Tarim Basin because they determine its endorheic/exorheic drainage. These complex climatic-drainage-tectonic interactions make the development of steady-state topography at the continental scale unlikely.

## Introduction

The surface of the Earth’s continents is primarily shaped by a competition between the relief-generating tectonic deformation and the relief-reducing erosion and surface transport of rock. As topography grows above sea level (or above a regional geomorphological base level) by tectonic motions, so do the energy, transport capacity, and efficiency of most erosion and surface transport mechanisms. In the continental scale, the main transport agent for surface sediment transport is the river network. While the combined effects of tectonics and erosion on the topography of continents have long been studied, only in the last 3 decades have numerical models provided some quantitative understanding of classical geomorphological concepts such as base level, retrogressive erosion, river piracy, or peneplanation.

More subtly, models of orogenic growth have shown that surface mass transport on the surface of the solid Earth not only responds to the tectonic deformation to shape geomorphological features, but it also actively modifies the distribution of stress within the crust and the subsequent tectonic deformation patterns. Field evidence for this phenomenon remains scarce [1–3], but analogue and numerical modeling techniques consistently predict that syntectonic erosion affects the localization of deformation within the orogenic wedge by influencing on the timing of fault activation and viscous creep in the crust [4], [3], [5], [6]. Surface transport has also been proposed to prevent the collapse of an intracontinental range as removal of material from topographic heights and deposition in the foreland oppose to spreading of the crustal root [7], [8]. In addition to the effects of erosion, the load of sediment in foreland basins formed in front of orogens induces forward propagation of thrusting [9–13].

This feedback has been addressed generally through 2D models (in cross section) and at the local and orogenic scales, whereas river networks drive surface transport typically parallel to the geological structures and far from the collision area. The question remains as to whether the asymmetrical fluvial transport may have an influence on lithospheric deformation [13] and how does the interplay extend to the continental-scale. Thin-sheet viscous models account for the basic trends of continental-scale deformation by considering the lithosphere as a viscous layer [14], [15] with vertically-averaged rheology and subject to plane stresses. Imposing lateral variations in lithospheric strength and forces and velocities acting at the boundaries of the thin sheet has allowed reproducing the crustal thickening observed in settings as diverse as the India—Asia collision (e.g., [16–19]; and references therein), western North America [20], north-eastern Brazil [21], the Iranian plateau [22], or central-western Europe [23]. [24] and [25] combined two respective thin-sheet codes with surface diffusive transport of sediment to study the Australian Palaeozoic lithospheric deformation and the Cenozoic growth of the Alps. [26] combined for the first time a thin sheet simulation of the growth of the Tibetan plateau with a stream-power erosion model, although this study focuses more on the effects of tectonics on drainage rather than looking at the two-way feedbacks between lithospheric and surface processes.

Separately, a growing view has developed that sees tectonic uplift and topographic changes (influencing the localization of rock weathering and erosion) as controls on the atmosphere and ocean circulation and therefore as global climate drivers (Ref. [27] and references therein). (U-Th)/He data show that erosion rates can be extremely localized by the orographic effects on precipitation [28], [29], and therefore orography may be a key player in determining the climatic effects on tectonics.

**Fig 1:**
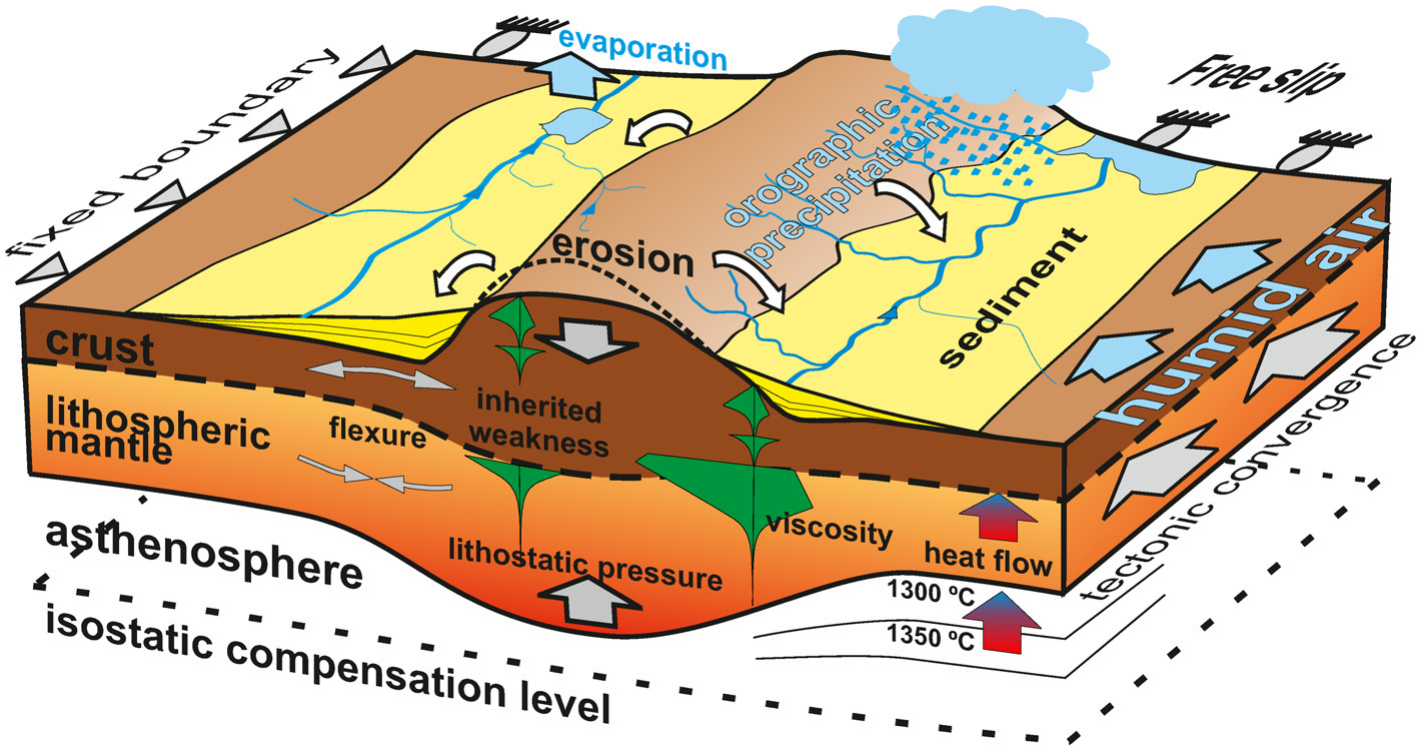
Conceptual cartoon with the processes integrated in the model. Not to scale. River erosion and sediment transport is calculated using a stream power approach and allowing the water follow the maximum slope. Evaporation is explicitly accounted for in lakes. The horizontal tectonic velocity is predefined at the boundaries. The viscosity of the lithospheric layer is dependent on the thermal regime and lithological parameters. The thickness of crustal and mantle varies according to the accommodation of the boundary velocities, depending on the viscosity distribution.

The purpose of this work is to evaluate the 3D continental-scale two-way interaction between lithospheric, and surface/climatic processes during continental collision by means of numerical modelling (Fig 1). For the first time, we simulate the feedbacks between orographic precipitation and inherited tectonic structures during the development of continental topography, using a model set-up inspired on the Cenozoic collision of India and Eurasia. With this tool, we aim at constraining the relative importance of each of the processes involved.

For this purpose, we develop a computer model that is novel in 1) incorporating a fully thermally-coupled formulation to a thin-sheet model of lithospheric deformation; 2) incorporating regional isostasy to a thin sheet deformation model to allow the formation of flexural sedimentary basins; 3) incorporating orographic precipitation; and 4) accounting for evaporation and climate continentalisation as mechanisms potentially leading to the formation of closed, endorheic lakes acting as surface water sinks and sediment traps. The source code of the programs solving the equations involved is publicly available to allow the reproducibility of the results. We now present a comprehensive description of the algorithms and formulations of the physical processes that we consider responsible for the main trends of the continental-scale topographic evolution.

## Formulation and Numerical Model

The developed computer program is named *UhuruTISC* after the two independent codes from which it derives: the thin-sheet thermomechanical model *Uhuru* [30] and an improved version of the surface process and flexural isostasy model *TISC* [31]. The formulation used for each of the processes considered is detailed in the following subsections. Here, we summarize the strategy adopted for each of them. The model fully couples the thermo-mechanics of a viscous thin-sheet submitted to horizontal forces with the dynamics of erosion and transport performed by the fluvial network and the uneven distribution of precipitation and evaporation imposed by the topographic evolution. Both horizontal (thin-sheet) and vertical (regional isostasy) motions are solved in 2D (planform view) disregarding the sphericity of the Earth. The model therefore accounts for the mutual effects between atmospheric humidity flow and surface sediment transport (both dependent on the evolving topography), and the viscous deformation and thermal evolution of the lithosphere (for instance, the sediment thermal blanketing or the erosional rise of the crustal isotherms).

The equations are numerically solved using the finite difference technique, using a fixed rectangular mesh and a time-explicit scheme. The code is written in C and Fortran for a Linux platform.

The model can be described as the combination of the following 6 components:

### Viscous thin sheet deformation

Viscous thin-sheet models are used to reproduce the main trends of the continental-scale development of topography and crustal thickness during collision. To calculate the lithospheric deformation we take a viscous thin sheet approach with vertically-averaged viscosity [14], [32]. Stiff and weak predefined zones determine where is the tectonic shortening accommodated as crustal/lithospheric thickening. We explicitly introduce these variations as a result of different activation energy for rock creep, accounting for the strong temperature dependence of viscosity [33], [18] and quantitatively linking the stiff/weak areas to lateral variations of the thermal regime (e.g., [18]; [34]; [16]. This fully-coupled thermo-mechanical formulation is described in [25].

The horizontal velocity field of the thin sheet is calculated in response to prescribed velocity at the boundary (e.g. continental indenter) and the gravitational potential energy related to lateral thickness and density variations of the sediment layer, the crust, and the lithospheric mantle [25]. The resulting velocity field is in turn responsible for the changes of the thickness of these 3 layers. The model thus accomplishes a mass balance: the amount of crustal material entering the model domain matches the crustal thickening in the orogen plus the sediment accumulation plus the sediment lost through the boundaries.

The topographic growth is simulated via the viscous deformation of a thin sheet representing the lithosphere, assuming mechanical coupling between crust and lithospheric mantle. The rheological behaviour of the thin sheet is depth-averaged, and hence the internal geometry of the tectonically deformed crust and sediments is out of the scope of this study. Because the typical scale for faulting is two orders of magnitude smaller than the orogenic or basinal features of our model, sediment is simply thickened in the same factor as the lithosphere does. At each time step and each cell of the model, the additional weight of rock related to the thin-sheet thickening and to erosion/sedimentation is added to the total load resting on the flexural plate.

Consider a thin viscous layer of thickness *H(x*,*y)*. We need to find the distribution of the mean horizontal velocity *u*_*1*_,*u*_*2*_ *(x*,*y)* and the correspondent thickening at each point of this thin sheet. Under the assumption of plane stress, where the vertical shear stresses are null, the deformation of the lithosphere can be treated in terms of vertically-averaged magnitudes. The momentum equations are vertically averaged within the plate, to reduce the three-dimensional problem to a planform model, where the horizontal velocity components do not change with depth (a detailed formulation is available in [25]).

#### Calculation of the tectonic velocity field

The equilibrium equation for the lithosphere is given, written compactly, by

$$\frac{\partial \;\sigma_{ij}}{\partial \;x_{j}}\;+\rho \;g_{i}=0;\;i,j\text{ = 1, 2, 3},$$ (1)

Where *σ* is the stress, *ρ* is the density, and *x*_*1*_ and *x*_*2*_ are the horizontal position axes and *x*_*3*_ corresponds to the vertical position (*x*_*3*_ = 0 at present sea level and positive downwards), $\vec{g}\;=\;(0,\;0,\;g)$ is the acceleration due to gravity (*g* = 9.8 m/s^2^) and the total stress is

$$\sigma_{ij}=\tau_{ij}\;+\;1/3\;\delta_{ij}\sigma_{kk}$$ (2)

Where *σ*_*kk*_ is the sum of the terms of the diagonal of the stress tensor, *σ*_*kk*_ = *σ*_*11*_ + *σ*_*22*_ + *σ*_*33*_, and *δ*_*ij*_ = 1 if *i* = *j* and *δ*_*ij*_ = 0 if *i* ≠ *j*. The deviatoric stress, *τ*_*ij*_, is related to the strain rate and temperature through the constitutive equation,

$$\tau_{ij}\;=\;2\;\eta \;{\dot{\varepsilon }}_{ij}$$ (3)

where η is the effective viscosity and *ε*_*ij*_ is the strain rate tensor which relation with the velocity field *u*_*i*_ is ${\dot{\varepsilon }}_{ij}=\;\frac{1}{2}\;\left(\frac{\partial \;u_{i}}{\partial \;x_{j}}+\frac{\partial \;u_{j}}{\partial \;x_{i}}\right)$, where *i*, *j* = 1, 2, 3

The main approximations for the thin-sheet approach state that: 1) the vertical shear stresses are zero and therefore every column is locally supported and there is no lateral exchange of vertical forces ([14]). Note that in this study we will independently account for these vertical stresses via a flexural isostatic model responding to the lateral variations of thickening/thinning of the viscous thin sheet; 2) The deviatoric stresses are assumed to vanish beneath the lithosphere, so there is no shear stress on the base of the lithosphere and no vertical variations of horizontal velocity. These can be written as:

$$\sigma_{13}\;=\sigma_{31}\;=\sigma_{23}\;=\sigma_{32}\;=\;0\;\Rightarrow \;{\dot{\varepsilon }}_{13}\;=\;{\dot{\varepsilon }}_{31}\;=\;{\dot{\varepsilon }}_{23}\;=\;{\dot{\varepsilon }}_{32}\;=\;0$$

Under these conditions, the vertical component of the equilibrium Eq (1) yields,

$$\frac{\partial \;\sigma_{33}}{\partial \;x_{3}}=-\rho \;g$$ (4)

These assumptions allow the deformation of the lithosphere to be treated in terms of vertically-averaged magnitudes. Therefore, the momentum equations are vertically averaged along depth *x*_*3*_ within the plate.

Considering the Eq (4), the fluid incompressibility (${\dot{\varepsilon }}_{33}\;=\;-\left({\dot{\varepsilon }}_{11}\;+\;{\dot{\varepsilon }}_{22}\right)$), the relation between the total stress and the deviatoric stress (Eq 2), the constitutive Eq (3) and the relation between the strain rate and the velocity field, the horizontal components of the equilibrium Eq (1) results, written compactly, in

$$\frac{\partial }{\partial x_{j}}\left[\eta \left(\frac{\partial \;u_{i}}{\partial \;x_{j}}\;+\frac{\partial \;u_{j}}{\partial \;x_{i}}\right)\right]\;+\;\frac{\partial }{\partial \;x_{i}}\left[2\eta \;\left(\frac{\partial \;u_{1}}{\partial \;x_{1}}+\frac{\partial \;u_{2}}{\partial \;x_{2}}\right)\right]\;=\;\frac{\partial \;{\bar{\sigma }}_{33}}{\partial \;x_{i}}\;$$ (5)

where *i*, *j* = 1,2 are the horizontal components; *u*_*1*_ and *u*_*2*_ are horizontal components of the velocity vector and they are the two unknowns; and $\bar{\sigma_{33}}$ is the depth-averaged vertical stress over the plate to include the gravitational potential energy variations induced by lateral variations of the different layers thicknesses and densities (*ρ*):

$${\bar{\sigma }}_{33}(x_{1},x_{2})=\frac{1}{H}\;\int_{0}^{H}\sigma_{33}(x_{1},x_{2},x_{3})\;dx_{3}\;=\;\frac{1}{H}\;\int_{0}^{H}\left(\int_{\begin{array}{l} surface\\ \left(x_{3}<0\right) \end{array}}^{x_{3}}g\;\rho (x_{1},x_{2},x_{3})\;dx_{3}\right)\;dx_{3}$$ (6)

where *H* is the thickness of the plate, and it is the depth of compensation level (within the asthenosphere) plus the elevation (elevation>0 for topography and elevation<0 for bathymetry).

Our plate consists of five layers: water, sediments, crust, lithospheric mantle and asthenosphere with thicknesses *h*_*w*_, *h*_*s*_, *h*_*c*_, *h*_*m*_, *h*_*a*_, and densities *ρ*_*w*_, *ρ*_*s*_, *ρ*_*c*_, *ρ*_*m*_, *ρ*_*a*_, respectively. Therefore, the depth-averaged vertical stress is,

$${\bar{\sigma }}_{33}\left(x_{1},\;x_{2}\right)=\frac{1}{H}g\left[\left(\rho_{w}\frac{h_{w}^{2}}{2}\right)+\left(\rho_{w}h_{w}h_{s}+\rho_{s}\frac{h_{s}^{2}}{2}\right)+\left(\left(\rho_{w}h_{w}+\rho_{s}h_{s}\right)h_{c}+\rho_{c}\frac{h_{c}^{2}}{2}\right)+\left(\;\left(\rho_{w}h_{w}+\rho_{s}h_{s}+\rho_{c}h_{c}\right)h_{m}+\rho_{m}\frac{h_{m}^{2}}{2}\right)+\left(\left(\rho_{w}h_{w}+\rho_{s}h_{s}+\rho_{c}h_{c}+\rho_{m}h_{m}\right)h_{a}+\rho_{a}\frac{h_{a}^{2}}{2}\right)\right]$$ (7)

#### Thickness changes of the thin sheet

The obtained horizontal velocity field allows calculating the thickness changes of a layer of thickness (*h*)

$$\frac{dh}{dt}\;=\;{\dot{\varepsilon }}_{33}\;h$$ (8)

where *t* is time and *ε*_33_ is the vertical strain rate, calculated from the incompressibility condition:

$$\;{\dot{\varepsilon }}_{33}\;=\;-({\dot{\varepsilon }}_{11}\;+\;{\dot{\varepsilon }}_{22})=-\;\left(\;\frac{\partial \;u_{1}}{\partial \;x_{1}}\;+\;\frac{\partial \;u_{2}}{\partial \;x_{2}}\right)$$ (9)

where $\vec{u}=(u_{1},u_{2})$ is the horizontal velocity vector. Since in an Eulerian reference system (a fixed, non-deforming grid),

$$\frac{dh}{dt}\;\equiv \frac{\partial \;h}{\partial \;t}+\;\vec{\nabla }h\cdot \vec{u}$$ (10)

then the change in layer thickness is, from Eqs (8) and (10),

$$\frac{\partial \;h}{\partial \;t}={\dot{\varepsilon }}_{33}\;h-\;\vec{\nabla }h\cdot \vec{u}$$ (11)

The changes in sediment and crustal thicknesses at a given node of the Eulerian grid ∂*h*/∂*t* are calculated solving by finite differences the Eq (11). The lithosphere thicknesses variations are determined after solving the thermal equation (see next section).

### Thermo-mechanical model

The pattern of deformation of the lithosphere also depends on the distribution of gravitational potential energy within the plate and therefore on the temperature-dependent density variations of the lithospheric mantle and the asthenosphere. The average viscosity and the gravitational potential energy determining the evolution of deformation are computed as a function of the thermal distribution within the lithosphere, which is calculated in 1D for every column of the model [30]. The effective viscosity *η* in Eq (5) is calculated from the depth-integral of the yield stress envelope (the depth distribution of the lesser of the brittle and ductile yield stress), to reflect its dependency on temperature and strain rate. Rock heat production by natural radioactive decay is also accounted for, since it amounts to about 40% of the total heat released to the atmosphere by the continental lithosphere [35], has as strong influence on the temperature distribution and the viscosity at lithospheric scale.

Deformation occurs by frictional sliding or dislocation creep. At each depth, the yield stress is given by the lesser of the brittle and ductile strength [71], [72]. The frictional sliding is a function of depth *x*_*3*_, according to *τ*_*brittle*_ = *β x*_*3*_, where the brittle failure coefficient *β* is the yield stress gradient depending on the type of fault, the angle of fracture and on the pore-pressure. Ductile deformation is governed by the power law creep equation when the applied stresses are less than 200 MPa and by the Dorn law equation for larger stresses. Then, the ductile deformation is given by,

$$\begin{array}{l} \text{Power law}:\;\tau_{ductile}\;=\;{\left(\frac{\dot{\varepsilon }}{A}\right)}^{1/n}\;exp\left(\frac{Q_{P}}{n\;R\;T}\right)\;\tau \;\leq \;200\text{ MPa}\\ \text{Dorn law}:\;\tau_{ductile}\;=\;\sigma_{D}\;\left[1\;-\;{\left(\frac{R\;T}{Q_{D}}\;ln\left(\frac{{\dot{\varepsilon }}_{D}}{\dot{\varepsilon }}\right)\right)}^{1/2}\right]\;\tau \;>\;200\text{ MPa} \end{array}$$ (12)

where *A*, *Q*_*P*_, *n*, σ_D_, *Q*_*D*_ and ${\dot{\varepsilon }}_{D}$ are material constants depending on the rock-type, *R* is the gas constant and *T* is the absolute temperature. $\dot{\varepsilon }\;=\;{\left[\;\frac{1}{2}\;(\;{\dot{\varepsilon }}_{11}^{2}\;+\;{\dot{\varepsilon }}_{22}^{2}\;+\;{\dot{\varepsilon }}_{33}^{2}\;)\;+\;{\dot{\varepsilon }}_{12}^{2}\;\right]}^{1/2}\;$ is an effective strain rate given by the second invariant of the strain rate tensor.

#### Thermal equations

The temperature distribution is given by,

$$\frac{d\text{​}T}{d\text{​}t}=\frac{\kappa }{Κ}\;\vec{\nabla }(Κ.\;\vec{\nabla }Τ)+\frac{\kappa }{Κ}Η$$ (13)

where *κ* is the thermal diffusivity, *K* is the thermal conductivity, *H* is the volumetric radiogenic heat production, and $\frac{dT}{dt}=\frac{\partial \;T}{\partial \;t}+\vec{u}\cdot \;\vec{\nabla }T$ is the total derivative of temperature (T) with time *t* and, therefore, it includes the advective term dependent on the tectonic velocity $\vec{u}$. The thermal model is simplified by considering only the vertical component of heat conduction, and it is solved in 1D fixing the temperature at surface and at the base of the plate. The lithosphere—asthenosphere boundary is defined as the depth to a reference isotherm (*T*_*a*_), which is affected by thermal advection (deformation), thermal conduction, and radiogenic heat production. To simulate the adiabatic gradient on the asthenosphere, we have assumed that it has a high thermal conductivity (~100 W m^-1^ K^-1^). The change in thermal conductivity between lithospheric mantle and asthenosphere is also a dynamic boundary, which moves with the isotherm that defines the base of the lithosphere (*T*_*a*_) and occurs 20 km beneath this isotherm. For more details see [30].

Volumetric heat production measurements range between 0–0.02 μW m^-3^ for mantle rocks, 0.1–1 μWm^-3^ for lower crustal rocks, and 1–4 μWm^-3^ for upper crustal rocks, although depth variations of radiogenic heat production in continental crust are debated. See the values adopted in Table 1.

**Table 1:** Main parameters and symbols. The values correspond to the reference setup MS0.

Parameter				Symbol	Reference value	Units
**Geometry**		Initial elevation (flat)			35	m
		Model domain			6000x6000	km
**Indenter**		Velocity		*u*	50	mm/yr
**Viscosity**	**Power law creep**	Upper crust		*A*	2.5·10^−8^	MPa^-n^ s^-1^
	**Factor**	Lower crust			3.2·10^−3^	
		Lith. mantle			1·10^3^	
	**Power law creep**	Upper crust		*n*	3	-
	**stress exponent**	Lower crust			3	
		Lith. mantle			3	
	**Power law**	Upper crust		*Q*_*P*_	138	kJ mol^-1^
	**activation energy**	Lower crust			251	
		Lith. mantle			523	
	**Dorn Law creep**	critical stress		σ_D_	8.5·10^9^	Pa
		Activation energy		Q_D_	100	kJ mol^-1^
		critical strain rate		*${\dot{\varepsilon }}_{D}$*	5.7·10^11^	s^-1^
	**Lithosphere viscosity**	Continent		*η*	Limited to 10^21^−10^25^	Pa s
		Hard block (Tarim)			3 x Continent	
		Indenter (India)			10^25^ (rigid)	
**Density**		Water		*ρ*_*w*_	1030	kg m^-3^
		Sediment		*ρ*_*s*_	2200	
		Crust		*ρ*_*c*_	2800	
		Lith. mantle		*ρ*_*m*_	*ρ*_*a*_(1+*α*/2(*T*_*a*_ *-T*_*moho*_))	
		Asthenosphere		*ρ*_*a*_	3200	
**Thermal regime of the lithosphere**		Conductivity	Sediment	*Κ*	2.4	W m^-1^ K
			Crust		3.0	
			Lith. mantle		3.2	
			Asthenosphere		100.0	
		Heat production	Crust	*H*_*c*_	0.7e-6 · exp(-z/15e10)	W/m3
			Lith. mantle		0	
		Temp. base of lithosphere		*T*_*a*_	1300	°C
		Thermal expansion		*α*	3.5e-5	K^-1^
		Thermal diffusivity		*κ*	1e-6	m^2^ s^-1^
**Flexural isostasy**		Elastic thickness		*T*_*e*_	65	km
		Young modulus		*E*	7·10^10^	N m^-2^
		Poisson coefficient		*ν*	0.25	-
**Surface processes**		Erodibility	Bedrock	*k*_*b*_	2.E-8	m yr^-1^ Pa^-1.5^
			Sediment		2.E-7	
		River transport capacity		*K*_*cap*_	1000	kg m^-3^
		Lake/sea sedimentation length		*L*_*f*_ *_sed*	50e3	m
**Climate**		Wind velocity		*u*_*w*_	7	m s^-1^
		Wind azimuth			-30 (NorthWest)	deg. N
		Air humidity		*RH*	1	-
		Background precipitation (turbulence)		*α’*	350	mm/yr
		Lake evaporation (for *u*_*w*_ = 0)		*E*_*0*_	2000	mm/yr
		Temperature at sea level		*T*_*sl*_	8	°C
		Standard air temperature lapse rate		*Γ*	0.0065	K/m
		Ground temperature gradient			0.004	K/m
**Numerical calculus**		Nodes of horizontal discretization grid		*Nx*, *Ny*	201x201	-
		Lithospheric vertical discretization		*Nz*	900	-
		Atmospheric vertical discretization			100	m
		Time step for tectonics and isostasy		*dt*	250,000	yr
		Time step for surface transport and climate		*dt_efiros*	1,000	yr

### Flexural isostatic model

The formation of foreland sedimentary basins next to collisional orogens is primarily controlled by the bending of the lithosphere under the weight of the growing tectonic building [36], [37]. It will be shown later that neglecting such flexural isostasy strongly underestimates the accumulation of sediments within the continent. Accordingly, we calculate the lithospheric flexure in response to tectonic and surface mass transport (changes in thickness of the lithospheric mantle, crust, sediment, and water column). To calculate the flexural isostatic, we adopt a 2D (planform) thin-plate elastic flexural approach as in [31]. The isostatic compensation level is set at 400 km depth, well below the base of the lithosphere. Changes in the weight of the column above that depth are taken as the vertical load *q*(x,y) acting on the lithospheric plate.

The mass redistribution related to tectonic, surface, and hydrological processes are thus isostatically compensated via a 2D thin-plate flexural approach with an elastic thickness distribution *T*_*e*_ *(x*,*y)*. The expression used to calculate the flexural deflection *w(x)* is (e.g., [73]:

$$\Delta \left(D(x,y)\cdot \Delta w(x,y)\right)+\rho_{a}gw(x,y)=q_{i}(x,y)$$ (14)

Where Δ is the Laplace operator, D is the rigidity of the lithosphere, *w* is the flexural deflection (vertical motion), ***ρ***_***a***_ is the density difference between the underlying viscous asthenosphere and the fluid overlaying the system (air or water), and *q*_*i*_ is the load distribution (in our case the pressure lateral differences causing the isostatic disequilibrium). *x* and *y* are the horizontal coordinates equivalent to *x*_*1*_, *x*_*2*_ in the previous section. The equation can be developed [74] into:

$$\begin{array}{l} D\frac{\partial^{4}w}{{\partial x}^{4}}+D\frac{\partial^{4}w}{{\partial y}^{4}}+2D\frac{\partial^{4}w}{{\partial x}^{2}{\partial y}^{2}}\\ +2\frac{\partial D}{\partial x}\frac{\partial^{3}w}{{\partial x}^{3}}+2\frac{\partial D}{\partial y}\frac{\partial^{3}w}{{\partial y}^{3}}+\frac{\partial^{2}D}{\partial x^{2}}\frac{\partial^{2}w}{{\partial x}^{2}}+\frac{\partial^{2}D}{\partial y^{2}}\frac{\partial^{2}w}{{\partial y}^{2}}\\ +\nu \frac{\partial^{2}D}{\partial y^{2}}\frac{\partial^{2}w}{{\partial x}^{2}}+\nu \frac{\partial^{2}D}{\partial x^{2}}\frac{\partial^{2}w}{{\partial y}^{2}}+2\frac{\partial D}{\partial x}\frac{\partial^{3}w}{{\partial x\;\partial y}^{2}}+2\frac{\partial D}{\partial y}\frac{\partial^{3}w}{{\partial y\;\partial x}^{2}}\\ +2(1-\nu )\frac{\partial^{2}D}{\partial x\;\partial y}\frac{\partial^{2}w}{\partial x\;\partial y}\\ -F_{x}\frac{\partial^{2}w}{{\partial x}^{2}}-F_{y}\frac{\partial^{2}w}{{\partial y}^{2}}-F_{xy}\frac{\partial^{2}w}{\partial x\;\partial y}\\ +\rho_{a}gw=q_{i}(x,y) \end{array}$$ (15)

which in absence of lateral rigidity D variations and horizontal forces F reduces to:

$$D\frac{\partial^{4}w}{{\partial x}^{4}}+D\frac{\partial^{4}w}{{\partial y}^{4}}+2D\frac{\partial^{4}w}{{\partial x}^{2}{\partial y}^{2}}+\rho_{a}gw=q_{i}(x,y)$$ (16)

The rigidity *D* is related to the thickness of the elastic plate *T*_*e*_ (the equivalent elastic thickness of the lithosphere) by

$$D(x,y)=\frac{E{T_{e}}^{3}(x,y)}{12(1-\nu^{2})}$$ (17)

We use a Young Modulus of ***E*** = 7·10^10^ N/m^2^ and a Poisson coefficient of ν = 0.25.

### Erosion and surface sediment transport model

To calculate erosion and deposition on top of the dynamic topography generated by the processes described above, we adopt a modified version of an open-source numerical model of fluvial transport named TISC [11], [31]. A stream power-law formulation is developed in this subsection. This formulation is hybrid (*sensu* [38]), i.e., it limits both the detachment of bedrock (erodability) and the transport capacity of the river. This allows obtaining erosion and sediment transport rates based on the local river water discharge (determined by climatic processes, see below) and river channel slope (as imposed by tectonic deformation). As a river reaches the shore of a lake or the sea, sediments are distributed throughout the water body in all directions from the river mouth and deposited assuming a rate decreasing exponentially with the distance from the mouth. Sediment overfilling of lakes, together with erosion at the lake outlet, ensures that lakes behave in the model as transitory or ephemeral phenomena that tend to disappear after the tectonic forcing ceases, as widely recognised in nature [39]. This transport model is mass-conservative and works in steady flow: all eroded material in a time step is either deposited within the model or driven out of the model domain through the boundaries.

Studies of river incision and sediment transport have provided quantitative models of landscape evolution driven by surface water flow (e.g., [75]. These models have been useful to quantitatively understand the processes leading to the tectonic defeat of antecedent rivers [76], [77] and the formation of intramountain basins [60]. River diversion or defeat is controlled by the mean precipitation in the catchment, the rate of tectonic uplift across the river path, and the rock strength of the uplifting area. However, these models do not address the formation of lacustrine endorheic system upstream from the tectonic barrier and the water balance between collected runoff and evaporation at the lake’s surface. This is of key relevance here because by definition the transition from an intra-mountain basin to an internally-drained high plateau requires the evaporation at the basin to compensate the collected water runoff.

The approach developed here is based on that developed by [31] and implemented in an improved version of the program *TISC*, publicly available at the authors’ website. Water flows following the maximum slope on the 3D evolving topography (each cell has 8 surrounding neighboring cells), using a steady flow approach to calculate erosion and transport along river channels. This means that no transitory effects in water flow (such as floods) are accounted, and that at each time step, the precipitation in the entre model domain equals the evaporation in the endorheic lakes plus the water reaching the ocean or exiting the domain through the boundaries.

Erosion is approached as a power law function of basal shear stress *τ*_*b*_:

$$\frac{dz_{t}}{dt}=-k_{b}{\left(\tau -\tau_{c}\right)}^{a}$$ (18)

*z*_*t*_ is the elevation of the surface of the solid Earth relative to the present sea level, positive upwards. Note that the spatial coordinates *x*, *y*, *z* are respectively equivalent to *x*_*1*_, *x*_*2*_, -*x*_*3*_ in the previous sections. Both the erodability *k*_*b*_ and *a* are positive constants. We use a value of *a* = 1.5 [78], for which *k*_*b*_ has been estimated at 8·10^−6^ (Mesozoic limestone; [79] and 1.6 ·10^−4^ m yr^-1^ Pa^-1.5^ (Oligocene flysch; [80]).

Shear stress *τ*_*b*_ at the river bed can be approached as the product of water density ρ, the acceleration of gravity g, the mean water depth of the channel, and the slope of the water surface *S*, also known as hydraulic gradient [81]:

$$\tau =\rho_{w}\;g\;d\;S$$ (19)

where *d* is the flow depth, and *S* is the slope of water surface, assumed to be equal to the slope of the riverbed. The water flow over the riverbed is described by an empirical relationship relating water flow speed *V* with the hydraulic gradient *S* (Manning's formula):

$$V=\frac{\text{1}}{n}R_{h}^{{}^{\frac{2}{3}}}S^{{}^{\frac{1}{2}}}$$ (20)

where *V* is the average velocity (m s^-1^), *n = 0*.*05* is the roughness coefficient, and *R*_*h*_ is the hydraulic radius (m) of the river channel. The hydraulic radius is a measure of the flow efficiency of a river channel, and since channel width is significantly larger than channel depth, it can be approached as *R*_*h*_ *~ = d*.

The water discharge at any cell of the network (m^3^ s^-1^) must accomplish the continuity equation:

Q = W d V (21)

where the *W* is the width of the channel expressed in meters. We assumed a channel width proportional to the seaway depth by a factor 10 to 50 and the critical uplift rates obtained are sensitive to the adopted value by a factor 2.

For the calculation of landscape evolution and river incision we combine Eqs (18)–(21), with an empirical relationship for channel width derived from previous river channel studies (e.g., [82]):

$$W=k_{w}Q^{a_{w}}$$ (22)

where *a*_*w*_ = 0.5 is an empirically-determined, dimensionless constant and *k*_*w*_ = 1.2 is a value comparable to mountain rivers and from the Zanclean flood [80]. An expression is obtained equivalent to the stream power law [83–85], [38]:

$$\begin{array}{l} \frac{dz_{t}}{dt}=K\prime \;Q^{m}S^{n}\;\\ where\;K\prime =k_{b}{\left(\rho_{w}g\right)}^{a}{\left(\frac{n}{k_{w}}\right)}^{\frac{3a}{5}},\;m=\frac{3a(1-a_{w})}{5},\;n=\frac{7a}{10} \end{array}$$ (23)

which allows calculating the incision rate given its water discharge *Q* and the slope along the river channel of slope *S* with a formulation fully consistent with the seaway flow of the 1D model above (the same *k*_*b*_, *k*_*w*_, and *a* parameters are involved). *m*, *n* are dimensionless exponents. Note that this formulation predicts a concavity of river profiles in equilibrium of *m/n* = 3/7, in agreement with the range derived from mountain river incision studies [38], regardless of the value of *a*. A water discharge of 1000 m^3^/s and a slope of 0.1% yield an incision rate of 0.2 mm/yr using the parameter values listed in Table 1. Erosion along the fluvial network is calculated using the stream power in Eq (23), adopting a constant erodability value of *k*_*b*_ = 2·10^−8^ m yr^-1^ Pa^-1.5^. The rock eroded at each cell is added to the sediment transport load *q(x*,*y)* (kg s^-1^) that is passed to the lower cell along the drainage network.

The previous stream power law is classified as a detachment-limited transport model [38], because the amount of sediment carried by the river is limited only by the erodability of the bedrock in Eq (23). To capture the deposition in continental (subaerial) environments (river aggradation), we need to make this model hybrid. This implies defining a limit to the transport capacity of the river. For this we define an equilibrium transport capacity *q*_*eq*_ that is a also power function of the water flow energy [38], [86]:

$$q_{eq}=K_{cap}\;Q^{m_{t}}\;S^{n_{t}}$$ (24)

We calculate *q*_*eq*_ adopting *m*_*t*_ = *n*_*t*_ = 1. We then scale the erosion rate in Eq (23) by *(q*_*eq*_ *-q)/q*_*eq*_ to reflect the sediment saturation of the river as *q* approaches *q*_*eq*_. For *q>q*_*eq*_, all excess sediment load *q-q*_*eq*_ is deposited.

Similar formulations have been proposed to explain the incision along rivers and the transition to aggradation, such as the stream power model [83]; the unit stream power model [84]; the shear stress model [81]; and the saltation-abrasion model [85]. The differences are however not substantial for the purposes in this paper since they all reproduce the basic effects of base level changes, river captures, or the transition from incision to aggradation by a decrease in discharge or slope.

All equations are solved using the finite difference method, using the same rectangular grid as for the thin-sheet equations.

### Hydrological model

This model component is partly based on that developed in [11], [31]. We adopt a steepest descent approach in which water moves from one grid cell to another following the maximum slope direction in topography following a steady flow approach (i.e., no transitory flood effects, the water precipitating at any given time step equals the water delivered to the hydrological sinks: sea and lake evaporation). Lakes form at local topographic minima, they loose water by evaporation proportionally to their surface area, and their water excess, if any, is delivered through an outlet river. Lakes can in this way become closed (internally-drained, endorheic, i.e., having no outlet) if they are large enough to evaporate all the incoming water. For the sake of simplicity, we include the amount of ground evapotranspiration as part of the local water runoff, which we hereafter refer to as *precipitation*.

Each topographic cell has a certain water discharge as a result of the addition of local runoff (rainwater delivered to the drainage network) and water coming from the tributary surrounding nodes. The resulting discharge is transferred to the lower node of the river (determined by the maximum slope) after evapotranspiration has been subtracted. Underground flow is ignored. The water balance in a cell located at *x*,*y* can be written as

$$Q\left(x,y\right)=\;\sum_{tributaries}discharge\;+\;\left(P\left(x,y\right)-E\left(x,y\right)\right)\;*\Delta x*\Delta y$$ (25)

Where *Q* is the local water discharge, *P* is rain precipitation (the component collected as surface runoff), *E* evaporation, and *Δx*, *Δy* are the dimensions of the cell. Conservation of total water flowing all over the model at a given time must also be accomplished:

$$\sum_{all\_cells}\left(P\left(x,y\right)-E\left(x,y\right)\right)*\Delta x*\Delta y\;=\;\text{Q drained to the sea + Q through boundaries}$$

We chose the climatic parameters to predict a precipitation distribution similar to the present one in the India-Asia collision zone and to best account for the present mean discharge of the main rivers. The code tracks precipitation waters towards enclosed lakes situated at local topographic minima of the basins and calculates the extension of these lakes accounting for surface evaporation that, when exceeding the amount of collected water, triggers endorheism by cancelling the lake outlet. Transitory flow effects are not accounted for, and the amount of water leaving the model by evaporation or along the boundaries equals the total amount of precipitation at every time step.

A specific feature of this surface process model is that the formation of lakes in local topographic minima is explicitly accounted for. The motivation behind this is the aim at capturing the key role of the geometry of lakes during drainage evolution, since they determine the localization of intramountain sedimentation in the water body and of erosion along the outlet. Lakes are geomorphologically important because they redefine the local base level that shapes the distribution of erosion. In practice, we first calculate the drainage network including the extension of lakes, ignoring the potential lake closure and reduction caused by evaporation. We do this by tracking all nodes in the grid from the lowest to the highest in elevation, which allows an efficient detection of the local topographic minima.

To account for endorheic basin formation, lake evaporation has been incorporated in the model as an improvement on the algorithm of [31]. The amount of lake evaporation is subtracted from the result to the lake output discharge. In our model, lakes are at water balance between inputs (precipitation, river discharge) and outputs (evaporation, outlet discharge), adopting a steady flow approach and dismissing underground flow. Thus, for an open lake,

$$Q_{out\_of\_lake}=\int_{\begin{array}{l} basin\\ area \end{array}}\text{P(x,y) }dx\;dy\;-\;\int_{\begin{array}{l} \text{lake}\\ \text{area} \end{array}}\text{E(x,y) }dx\;dy$$ (26)

where *P* is the mean precipitation rate in the lake’s hydrographic basin of area *A*_*lb*_; *E* is the evaporation at the lake’s surface; and *A*_*l*_ is the area of the closed lake. The level of lakes changes as a function of lake evaporation *E*, precipitation runoff *P*, and river supply *R*.

Evaporation can eventually cancel the lake's water excess (see Eq 26) and then reduce its level below the outlet, causing the closure of the basin. From a technical, programming, and algorithmic point of view, this is probably the most complex component of the surface processes involved in the present study. We solve it by tracking all the cells in the topographic grid from top to bottom, transferring water discharge following the drainage network as previously defined, and removing from the closed lakes the highest cells of lakes until the two right-hand terms in Eq (26) are approximately balanced. Because the highest cells of a lake are the outlets, if evaporation is large enough the lake will loose all the outlets and become endorheic (closed drainage). When removing further nodes from the endorheic topographic basin, the lake may become split in several local topographic minima that will become isolated lakes with independent water balance. The level of a closed lake in steady flow is the one balancing water inputs and outputs, which can be written as

$$\sum_{\begin{array}{l} \text{lake}\\ \text{basin cells} \end{array}}\text{P(x,y) }\Delta x\;\Delta y=\sum_{\begin{array}{l} \text{lake}\\ \text{surface cells} \end{array}}\text{E(x,y) }\Delta x\;\Delta y$$ (27)

If the water level of a closed lake rises higher than the lowest surrounding topographic saddle (for instance, due to erosion of the surrounding topography or to a rise in water level caused by higher water discharge into the lake), then the drainage becomes open. Normally, incision along the outlet river will lead to a relative fast integration of the lake into the drainage network (drainage capture).

### Climatic model

Before calculating water discharge and river incision, the model needs to estimate the precipitation and evaporation distribution throughout the evolving topography. We have developed an algorithm based on a previous cross-section model by [40]. To account for the topographic dependence of precipitation and evaporation, we implement the flow of air with vertically-averaged humidity over the entire model domain. This wind flow is assumed to have a constant *x*,*y* direction while the air column moves vertically following the local topography. Humid air enters the model through the upwind boundaries of the model domain and then follows the mean wind direction (assumed constant in this paper). The air column changes in water content by exchanging water with the surface of the solid Earth via condensation (precipitation) and evaporation, both controlled by the level of water saturation of the air column. As the air column is forced to move uphill, it reduces its capacity to contain vapour, eventually losing water by precipitation. Also over flat regions the air produces precipitation by turbulent flow. In this way the model captures the gradual continentalization of weather (aridification) as air moves inland. The content of water in the air column only increases by evaporation, when passing over the sea or a lake. The model is water conservative: the amount of atmospheric water entering the domain through the boundaries equals the amount of water leaving it either as air moisture or as surface water, for each time step of the model evolution.

The orographic precipitation and evaporation module is a pseudo-3D adaptation of the pseudo-2D (cross-section) model developed in [40] to parameterize the interaction between erosion and tectonics during the build-up of high-plateaus. The model consists of a water-conservative air flow from side to side of the model domain, with a laterally and temporally constant air velocity. The moisture of the air is then decreased by precipitation or increased by evaporation over lakes and oceans. The rates of precipitation and evaporation are controlled by the level of water saturation of the air. The maximum water content in an air column $H_{W}^{\text{max}}$ (measured in m) can be derived from the Clausius-Clapeyron equation (e.g., [87]:

$$H_{w}^{max}\left(x,y\right)=\int_{z_{t}}^{\infty }\rho_{v}\left(z\right)dz=\int_{z_{t}}^{\infty }\frac{e_{s}\left(z\right)}{R_{v}T\left(z\right)}\;dz=\int_{z_{t}}^{\infty }\frac{e_{s0}}{R_{v}T\left(z\right)}\text{ exp}\left(\frac{L}{R_{v}}\left(\frac{1}{T_{0}}-\frac{1}{T\left(z\right)}\right)dz\right)$$ (28)

where *z*_*t*_ is the ground elevation, *ρ*_*v*_ is the vapor density, *e*_*s*_ is the saturation vapor pressure at a given temperature *T*, *R*_*v*_ = 461.5 J kg^-1^ K^-1^ is the vapor gas constant, and *e*_*s0*_ = 611 Pa is the saturation vapor pressure at *T*_*0*_ = 273 K. Air temperature varies with altitude above the ground (*z-z*_*t*_) as

$$T(x,y,z)=T_{zt}-\Gamma \cdot \left(z-z_{t}\right),\text{ where }T_{zt}(x,y)=T_{sl}-.004\cdot z_{t}(x,y)$$ (29)

where *T*_*zt*_ is the ground temperature (at *z = z*_*t*_), *T*_*sl*_ is the ground temperature at sea level, in K, and *Γ* = 0.0065 K/m is the standard temperature lapse rate in the atmosphere. As a result, the maximum water content in an air column $H_{w}^{max}$ is typically in the range of 0.05 to 0.2 m.

The actual water content in an air column *H*_*w*_ (m) changes as a result of the incoming humid air, plus the evaporation supplied from the surface minus the precipitation. Vapour mass conservation provides the spatial derivative of *H*_*w*_:

$$\frac{dH_{w}\left(x,y\right)}{dl\prime }=-\frac{P(x,y)}{u_{w}}+\frac{E(x,y)}{u_{w}}$$ (30)

where *dl’* is a distance differential in the wind direction, *P* is the precipitation, *E* the evaporation, and *u*_*w*_ is the absolute value of the mean horizontal wind speed. Such approach captures the vapor mass balance between the incoming wind and the evaporation/precipitation exchange with the ground, which has been neglected in previous surface process models but is of capital importance in this paper because humid air is expected to cross a series of topographic highs and lows containing lakes that reload the air with moisture.

Atmospheric water precipitation results mostly from condensation by quasi-adiabatic air decompression and cooling during air uplift. In turn, air uplift takes place mainly in two ways: by encountering an orographic obstacle (the vertical shifts imposed by topography cause *H*_*w*_ to approach or retreat from the saturation value $H_{w}^{max}$) or by local turbulent flow. These two mechanisms are captured in the following expression:

$$P\prime (x,y)=\alpha \frac{H_{w}(x,y)}{H_{w}^{max}(x,y)}$$ (31)

where *P’* is the condensation produced by the air column above any given location *x*, and *α’* [L T^-1^] is a constant controlling the precipitation occurring due to turbulent air flow when saturation is not reached. Processes such as air uplift after a humidity reload over a lake or an ocean may result in $H_{w}>H_{w}^{max}$ (saturation) and then $H_{w}-H_{w}^{max}$ is instantaneously dumped as condensation *P’*. Note that the orographic effect on condensation is contained in $H_{w}^{max}$, since this strongly decreases with altitude. The effect of continentality is captured because *H*_*w*_ is reduced by precipitation as air moves inland. The adopted boundary condition is $H_{w}\;=\;H_{w}^{max}$ ·*RH* (*RH* is the relative humidity of the incoming wind, see Table 1) and therefore precipitation in the boundary is *α·RH*.

We implement lateral smoothing along the upwind direction of this orographic precipitation [88], related to non-instantaneous water condensation, local turbulence, and deviations from the mean wind velocity adopted values. Using a Gaussian smoothing function the precipitation *P* at a given location is:

$$P(x,y)=\frac{2}{\Delta l\;\sqrt{\pi }}\;\int_{\begin{array}{l} l\prime =x,y\\ along\;wind \end{array}}^{\infty }P\prime (x\prime ,y\prime )\;e^{-\frac{{\left(x\prime -x\right)}^{2}+{\left(y\prime -y\right)}^{2}}{\Delta \;l^{2}}}dl\prime$$ (32)

where *Δl* is the smoothing distance, typically within a few km. Due to the large scale of the domain in the present work, this parameter is not influential.

Previous computer models of long-term surface transport did not account for evaporation. Whereas evapotranspiration along the river network can be easily accounted for as a reduction in local runoff or precipitation, evaporation at the surface of lakes must be explicitly incorporated if closed drainage basins are to be generated. Here an approximation is adopted according to which evaporation increases linearly with wind speed (similarly to [87]) and with *A*_*max*_-*A*:

$$E\left(x,y\right)=E_{0}\left(1+\beta \;uw\right)\frac{H_{w}^{max}-H_{w}}{H_{w}^{max}}$$ (33)

Where *E*_*0*_ is a reference evaporation rate for a hypothetical dry, calm air (*H*_*w*_ = 0, *u*_*w*_ = 0), and *β* is the proportionality constant with wind velocity.

## Reference Model Set-Up

**Fig 2:**
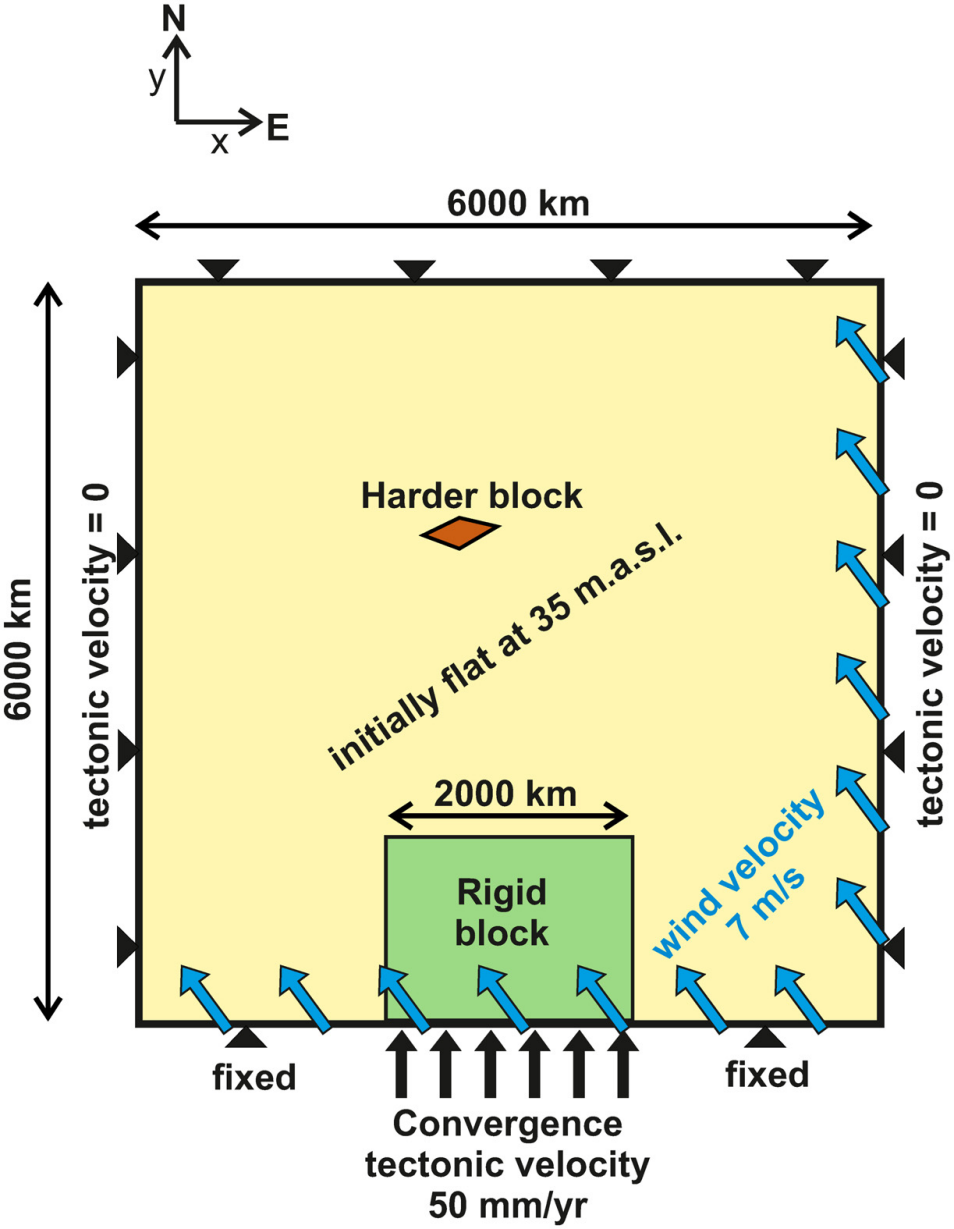
Model set-up and other boundary conditions. The tectonic boundary conditions (black) consist of a 50 mm/yr indentation of a rigid block at the southern boundary and fixed (*v*_*x*_ = *v*_*y*_ = 0) elsewhere. Vertical isostatic motions are allowed at the entire boundary (zero slope and zero momentum across the boundary). The harder block in red has 3 times higher viscosity than the rest of the model domain. The orographic precipitation model is controlled by a constant velocity and direction of incoming humid air (blue arrows). The initial topography is set flat and 35 m above sea level, with an additional random noise between ±5 m. In the text, the positive-*y* direction is referred to as ‘north’, and the positive-*x* direction is named ‘east’.

For the initial set-up of the reference model (MS0; parameters in Table 1), we predefine an initially flat continent at an elevation of 35 m, with a 32 km-thick crust and a base of the lithosphere located at a depth of 133 km. The boundaries of 3 distinctive domains in this continent are inspired by the India-Eurasia collision (Fig 2): 1) Indenter (completely rigid, representing India); 2) An intracontinental rigid domain (with a vertically-averaged viscosity 3 times higher than the rest of the model domain, representing the Tarim block); and 3) The rest of the model domain, representing Asia.

**Fig 3:**
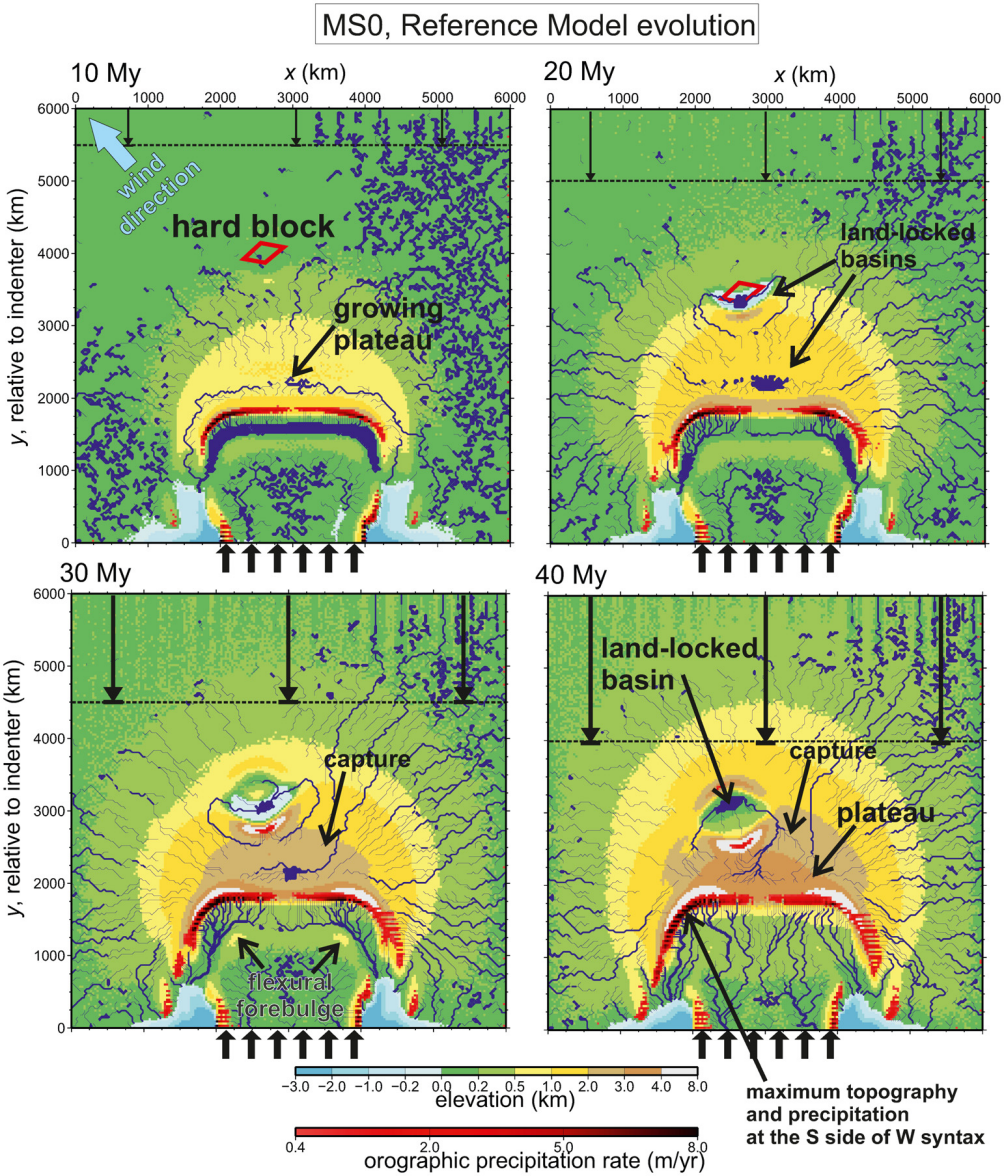
Topographic evolution of the reference setup MS0 at t = 10, 20, 30, and 40 Myr. A complete animation of the topographic evolution is available in the multimedia repository. Red shading shows areas where precipitation is higher than 0.4 m/yr. Note that the y-direction axis is the location relative to the indenter, so the rigid block appears to move southwards. Note the changes in drainage connectivity (captures) promoted by the tectonic growth of the plateau. The early endorheic plateau is captured by a northern river after 24 Myr which is in turn captured by the Tarim-like closed basin at t = 36 Myr, favoured by the lack of thickening of that block and its strong isostatic subsidence of over 8 km.

**Fig 4:**
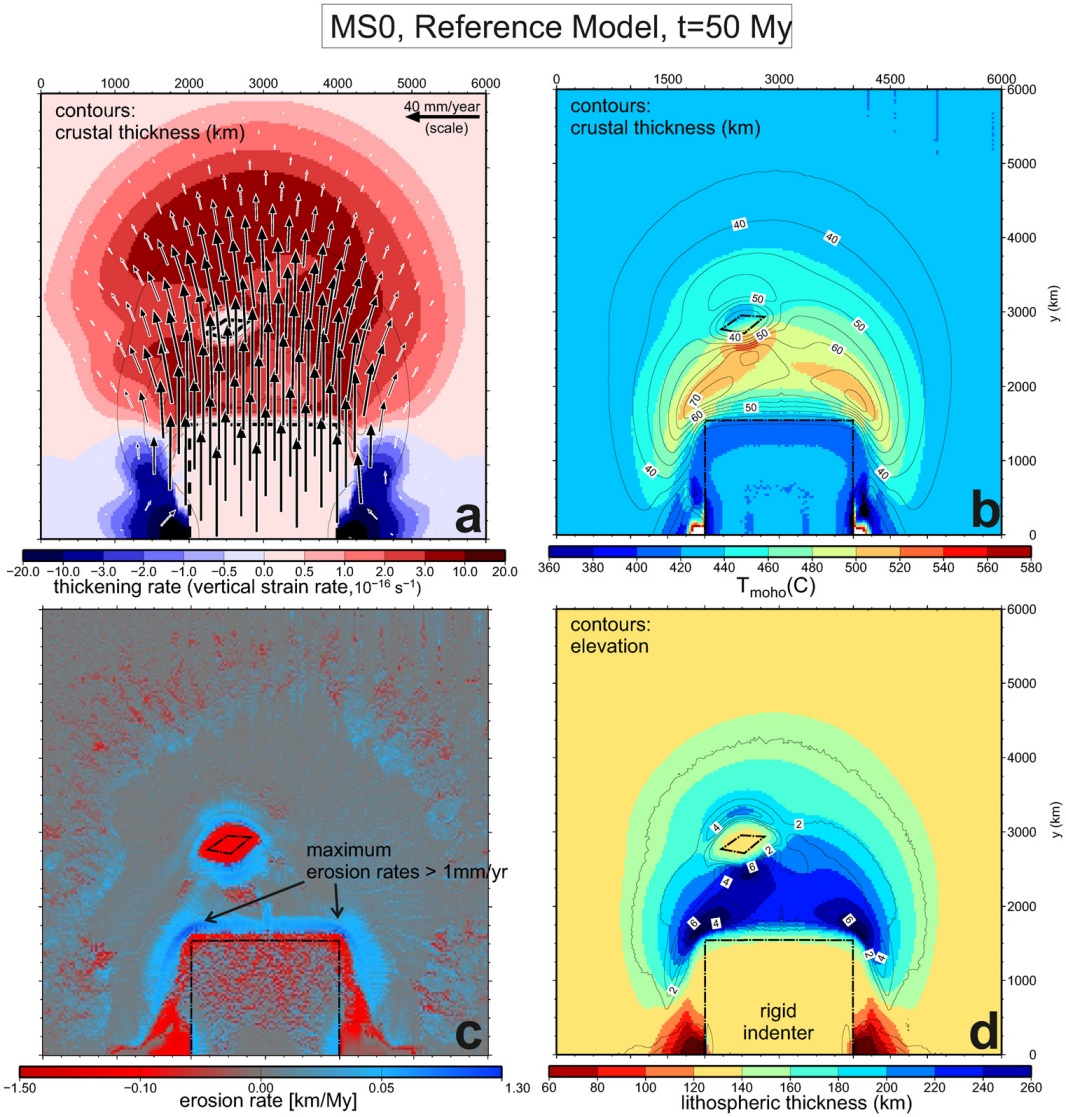
Final stage (*t* = 50 My) of the reference model setup MS0. The planform distribution is shown of a) rock velocity relative to the undeformed continent and vertical strain rate (thickening rate); b) Temperature at the base of the crust (shade) and crustal thickness (contours); c) erosion and sedimentation rate (maximum erosion rates are attained at the two syntaxes formed at the two corners of the indenter); and d) lithospheric thickness. The hotter base of the crust in thickened areas (as the plateau) causes weakening and further concentration of deformation in those areas. This in turn leads to the maximum topography in the syntaxes formed at the corners of the indenter (Fig 3). The southern side of the western syntax faces the wind direction (i.e., the topographis slope is opposite to the wind direction) and therefore concentrates more precipitation, leading to the highest erosion rates in the model, above 1 mm/yr.

For computing-efficiency reasons (to keep the modelling domain small and around the area of deformation), the modelling domain is shifted northwards together with the indenter (technique used also in [19]). Thus, in the planform views in Fig 3 and in the S1 video the continent appears to move southwards, while the velocity arrows at the indenter and at the growing plateau (Fig 4) have a northward direction. Keeping this in mind, the upper, right, and left boundaries of the model are locked (null velocity), while the indenter moves towards the north at 50 mm/yr. While the model setup could be easily adapted to reproduce more closely the Himalayan collision and the formation of the Tibetan Plateau (for instance, the right boundary could allow the exit of rock to reproduce the eastwards lateral extrusion reflected in GPS measurements in the Tibet, [41]), we opt here for keeping the model simple and focus on identifying process interactions rather than on finding geodynamic implications for the Himalayas-Tibetan Plateau region. Keeping this in mind, we will refer to the positive *y* direction as North (N) and the positive *x* as East (E), and we will use the terms *Tarim* and *India* to refer to the rigid block and the indenter, respectively.

Although the model allows for lateral changes of the elastic thickness, for the reference setup we adopt again for simplicity a constant elastic thickness of 65 km, a compromise between values estimated for the Tarim (40–100 km; [42], [43]) and the Ganges basins (80–90 km) [44].

We impose a relative humidity of the incoming air of RH = 1, entering the model from the southeast with a 30-degrees azimuth wind direction.

Other climatic, erosional, and tectonic parameters are listed in Table 1.

## Results

The topographic evolution of the reference model is shown in Fig 3 and the S1 video, while Fig 4 shows details on the final stage at *t* = 50 My, which represents the present day if comparing with the India-Tibet analogue. Note that the origin of the *y* axis moves in Fig 3 with the indenter, so the rigid block and the rest of the undeformed continent appear to move southwards in this sequence. After 10 Myr (500 km) of convergence, the initially flat continent has developed a 3000x1000 ~km region at an elevation close to 1000 m in front of the indenter. This is consistent with the substantial surface uplift reported in the Tibet already during the Eocene [45]. This region is bounded to the south by an E-W trending, 1000–2000 m-high mountain range that is already inducing some localization of rainfall along its southern flank. The weight of this mountain range is also causing the flexural subsidence of the northern side of the indenter and creating a lacustrine depositional environment. By *t* = 20 Myr this foreland basin has become fluvial, drained by two main rivers towards both sides of the indenter, in a position similar to the present-day Indus and Ganges rivers. Deposition in this basin takes place by fluvial aggradation, as it will be seen later. Meanwhile, the hinterland of the range (halfway to the *Tarim*-like block) has become a large plateau with a large endorheic basin centred on a large closed lake at an elevation above 1200 m. The rest of this plateau drains towards the boundaries of the model except for its central northern part that drains towards another closed endorheic basin formed on the pre-imposed rigid block (equivalent to the ‘*Tarim*’ block). The little amount of precipitation collected by this basin, together with its high evaporation rates (both caused by the dry climatic conditions forming in this lee-side of the plateau), cause this ‘Tarim’ lake to remain small in area and below sea level. Note that all the endorheic lakes in the model acquire exactly the surface area needed to evaporate the water supplied by rivers. The lake system in the high plateau is later captured by the eastern rivers at *t* = 30 Myr, and eventually becomes captured too by the *Tarim* drainage (*t* = 40 Myr).

**Fig 5:**
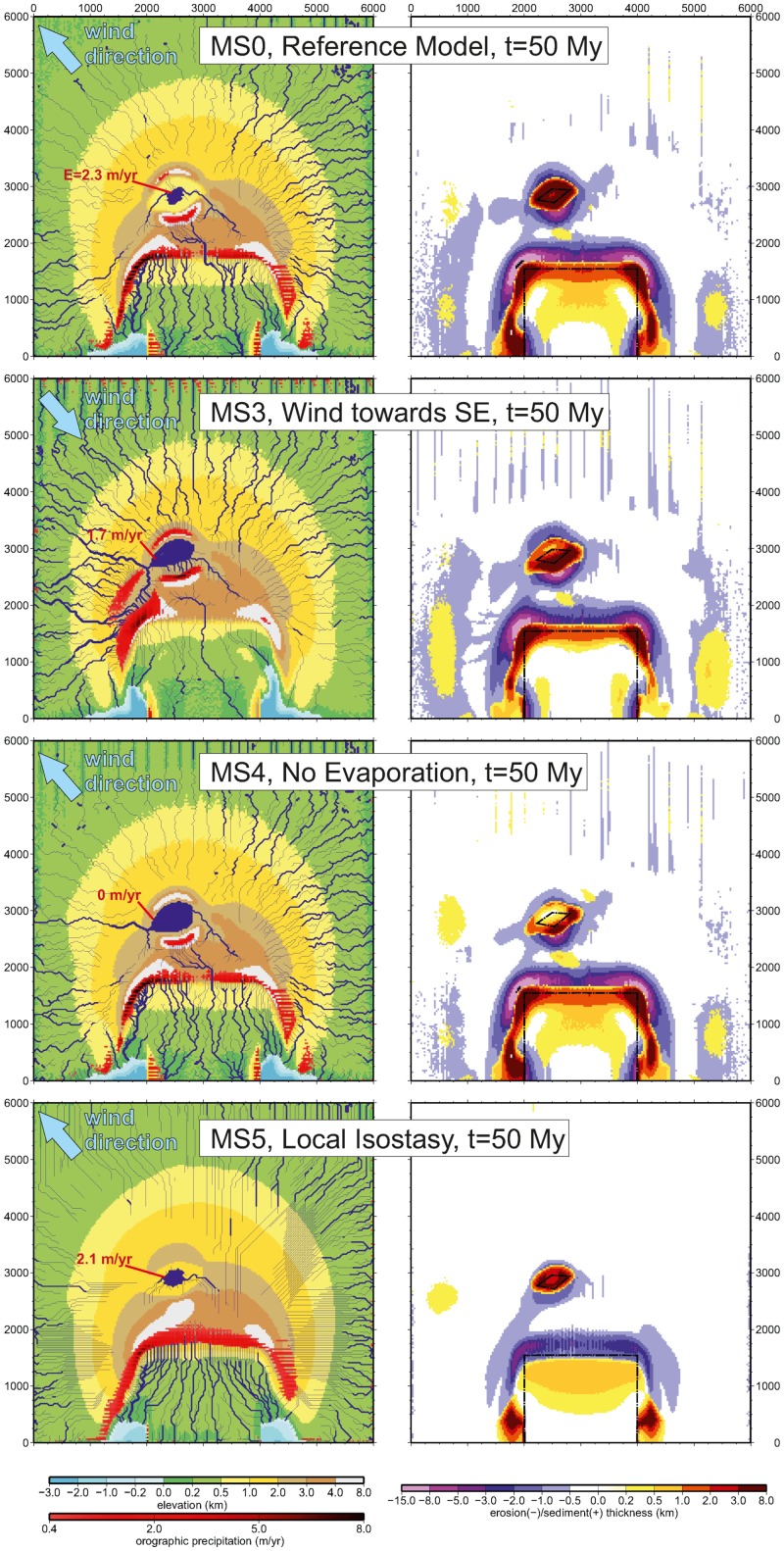
Effect of climatic parameters and isostasy on the final topography, precipitation, drainage, and sediment distribution. a) the reference orographic precipitation setting (reference setup MS0), b) changing wind direction (SE instead of NW, setup MS3). Numbers in red indicate lake evaporation rates. c) In absence of evaporation (MS4); and d) a model under local isostasy (*T*_*e*_ = 0; MS5). The red shading shows areas where precipitation is higher than 0.4 m/yr. The setups implying a wetter climate in the plateau region (MS3, MS4), show less sediment accumulation than the drier ones (MS0, MS5), because they inhibit endorheism and thus part of the sediments are drained out of the model boundaries. The absence of flexural isostasy in MS5 causes a smaller amount of sediment accumulation in all basins.

The final topography at *t* = 50 My, (Figs 4d and 5a) shows a relative similitude to the Tibetan plateau, with a 3000x1200 km region above an elevation of 3 km. The topography in this region is underestimated by about 1 km, but this was expected because processes such as lithospheric mantle removal have been previously pointed as responsible for a substantial part of the Tibetan Plateau elevation [19], [46], [47]. Again we have opted for not incorporating further complexity in our tectonic setting because we want to focus on the understanding of the process interplay rather than reproducing all the available observables of the Himalayan-Indian system. Another feature of the calculated topography that shows the limitations of our modeling approach is the elevation maximum, located in the southern flank of the Tarim block, instead of the southern flank of the plateau. Overall, the final topography is reflecting the large lateral variations of the thickened crust and lithospheric mantle, as displayed in Fig 4. The thickness of the crust and the lithosphere remains nearly unchanged (32 and 130 km respectively) in the most distal parts of the model domain and in the rigid *Tarim* block. In contrast, both thicknesses have more than doubled in the southern flank of the plateau, reaching maximum values of 80 and 290 km respectively in the syntaxes.

**Fig 6:**
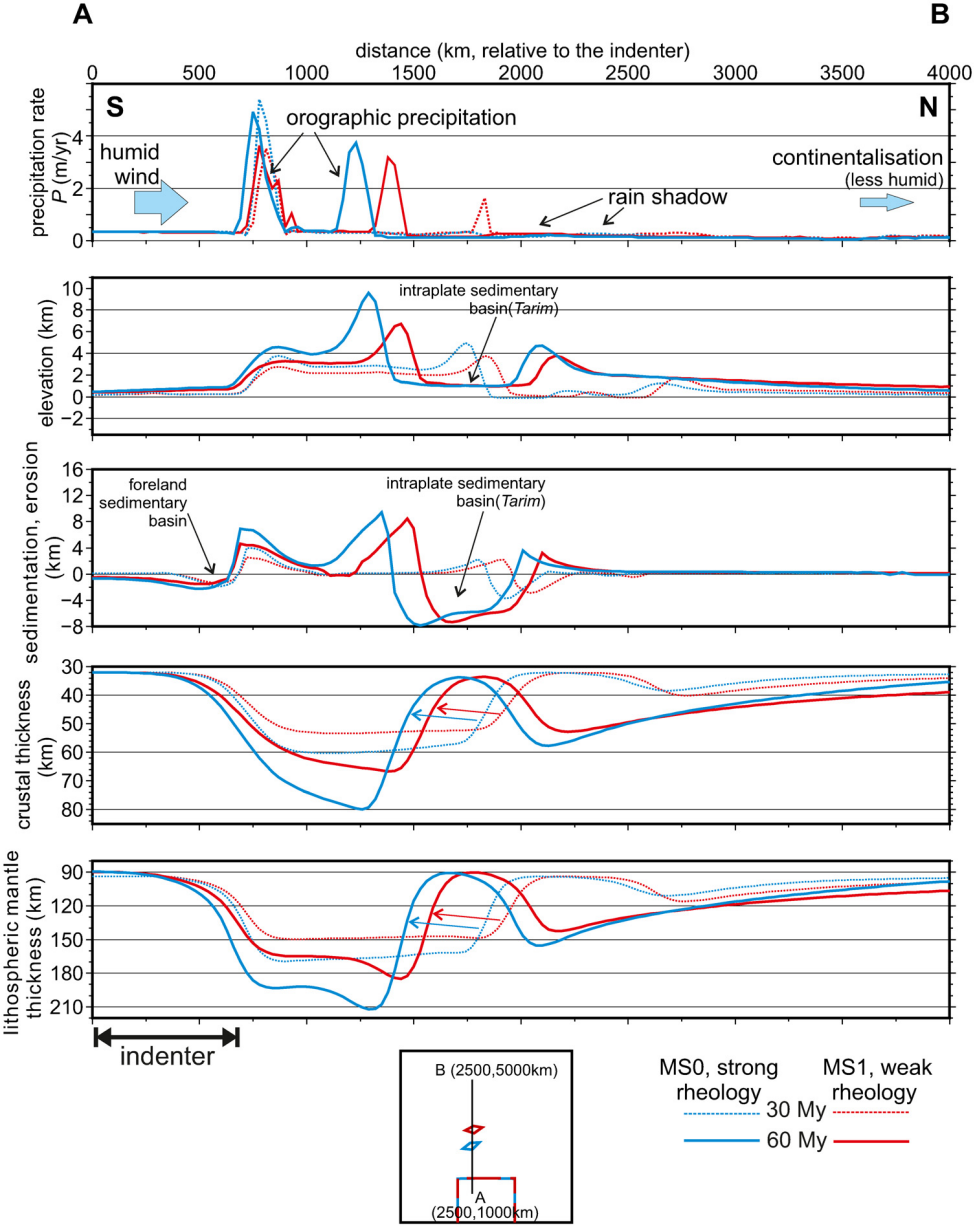
Effects of lithospheric heterogeneities on the topographic evolution. Profiles of precipitation, erosion, topography, sedimentary thickness, crustal thickness, lithospheric mantle thickness, for the reference model setup MS0 and for a weaker rheology (MS1), at 30 and 60 My. A weaker lithosphere implies a further propagation of deformation towards the interior of the continent, imposing a more distant position of the Tarim basin. This in turn induces larger orographic effects in the strong-rheology setup: higher upwind precipitation in the left, and more pronounced rain shadow to the right.

The lateral crustal and lithospheric contrasts cause in turn a differential isostatic adjustment via the flexure of the continent. Flexural basins form in areas of sharp contrast in lithospheric thickening (areas of little deformation near zones of intense thickening). One such area is the northern edge of the indenter. The foreland basin formed on the northern boundary of the indenter match to first degree the Cenozoic sedimentary thickness of the Indus and Ganges basins, with maximum values that change along the E-W strike from 2 to 7 km and a basin width ranging from 250 to 700 km (Figs 5a and 6). Another comparison that ensures that the surface processes are working at the right speed is the sediment thickness accumulated at the basin formed on the rigid block, ranging from 5 to 8 km (Figs 5 and 6), coinciding with observations compiled by [42]. Regional isostasy is responsible for most sediment accumulation within the continent (in both foreland or land-locked basins), by creating flexural basins next to areas of topography growth. Smaller sediment accumulation is predicted if the elastic thickness of the lithosphere is reduced (Fig 5). Also *T*_*e*_ values significantly larger than 65 km imply smaller sediment accumulation within the continent, because the *T*_*e*_ value adopted for the reference model optimizes the volume of accommodation space created by flexural isostasy.

**Fig 7:**
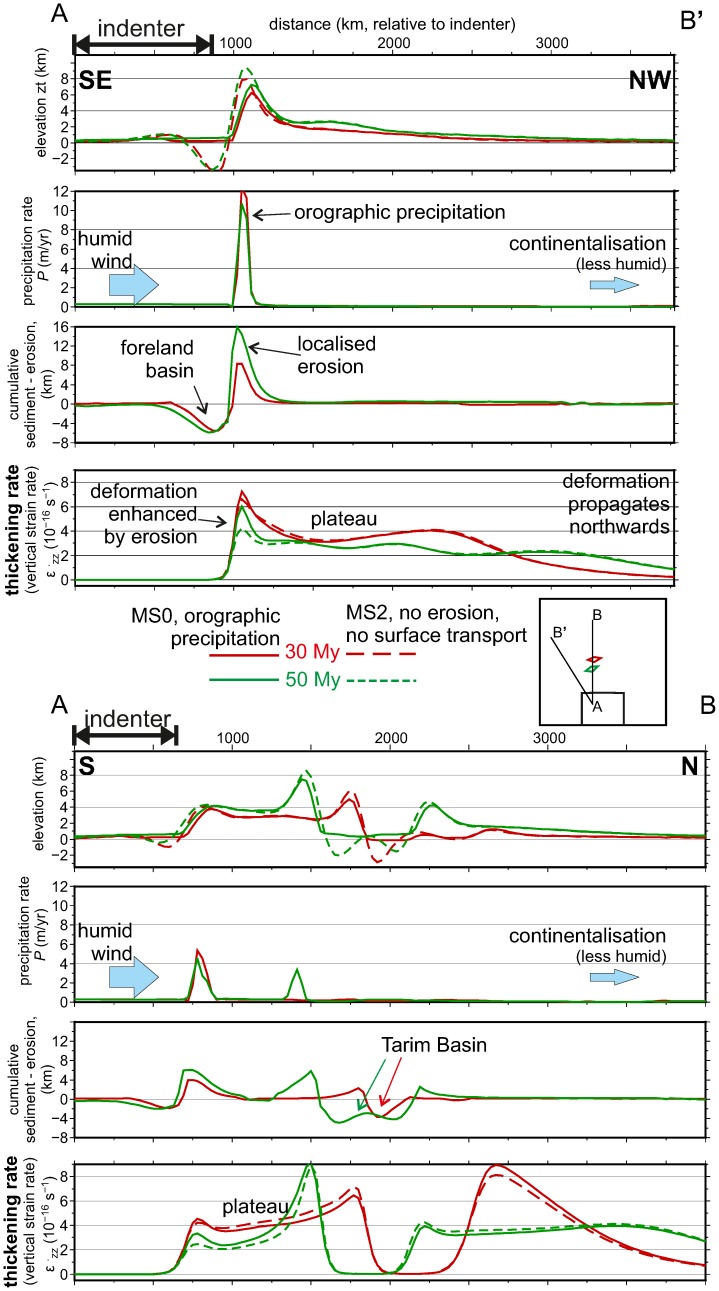
Effects of climate on tectonic deformation. Profiles of elevation, precipitation, cumulative erosion and sedimentation, and vertical strain rate (crustal thickening rate) for 2 stages of the reference setup MS0 (bold lines), and for a model with neither precipitation nor erosion (M2). A-B’) SE-NW profile along the corner of the indenter; A-B) S-N profile crossing the rigid block (*Tarim basin*-equivalent). Note that the orographic precipitation at the southern flank of the plateau is higher in the A-B’ section, because it faces the upwind direction.

The precipitation rates vary from >9 m/yr in the southern flank of the plateau to less than 0.1 mm/yr in the Tarim-like basin (Figs 6 and 7), within the order of magnitude measured in the Himalayan syntaxes and in the Tarim basin respectively. Although we adopt a constant wind velocity that extremely simplifies the monsoonal transport of atmospheric moisture in the vast region of northern India and central-eastern Asia (e.g., [48], the orographic precipitation model captures fairy well the contrasts between the lee and the upwind sides of the Himalayas. The temporal and spatial distribution of precipitation in response to topographic growth can also be appreciated in the profile A-B’ in Fig 7, which runs parallel to the wind direction.

**Fig 8:**
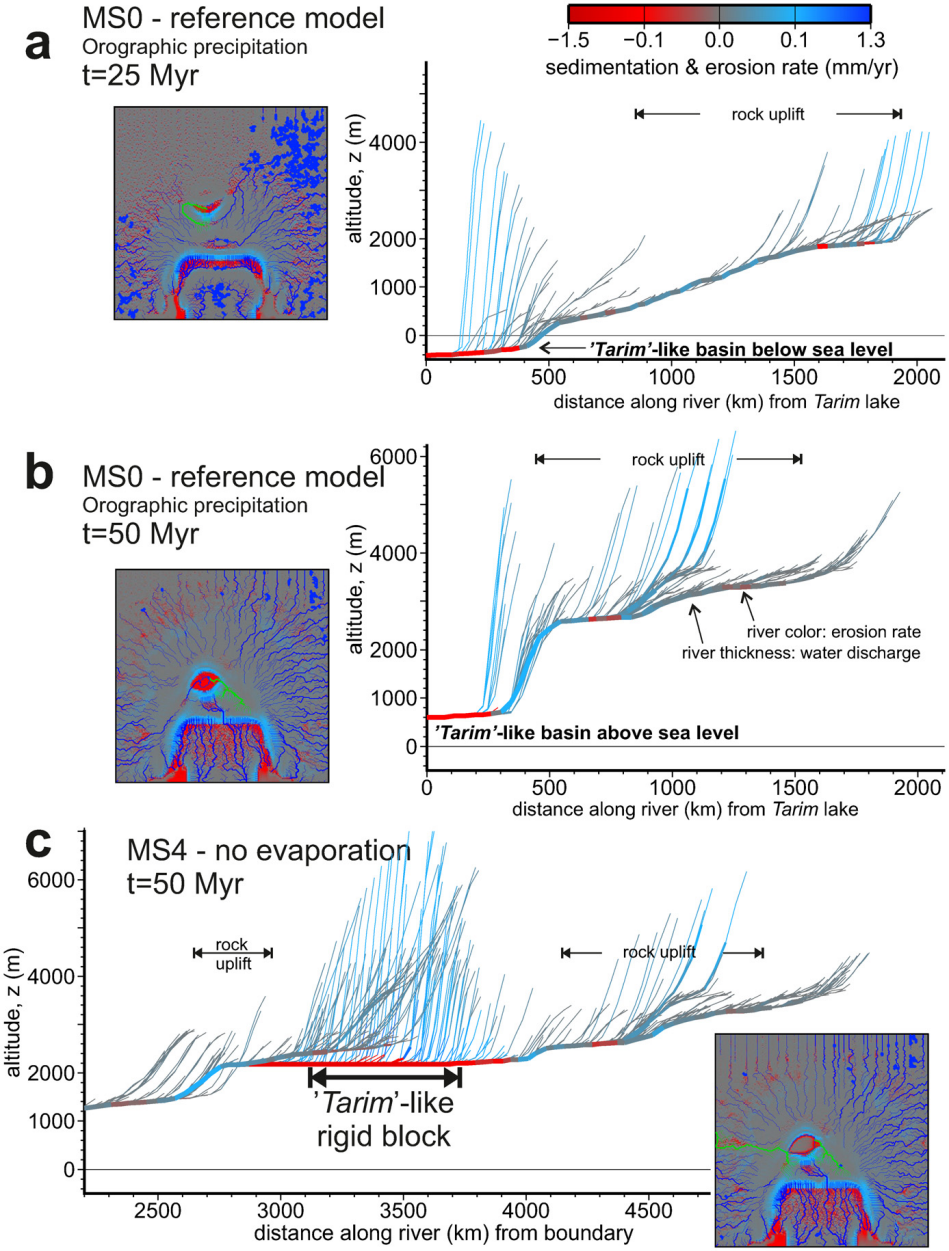
Elevation profile of selected river basins that drain the plateau towards the intracontinental *Tarim-like* basin. Two stages of the reference model setup are shown: a) 25 and b) 50 Myr (MS0). c) Shows the final stage of an identical setup disregarding lake evaporation (MS4). River pen size is scaled with water discharge. The colour of the river in the profiles indicates erosion (blue) and sedimentation (red) rates. The insets locate the river basin being displayed (in green) and show the distribution of erosion and sedimentation rates over the entire model domain (red and blue). Note that the river displayed in b results from a dramatic river capture of a N-draining river by a tributary of the *Tarim* basin (both visible in a). The *Tarim* basin turns a geomorphological base level for the plateau, and results 1300 m higher in MS4 (c) relative to MS0 (b).

In Fig 8 we show the elevation vs. distance profiles of three selected rivers at 25 My and 50 My that have in common that they drain towards the Tarim block. These river long-profiles record the tectonic and drainage evolution of their catchments. The evaporitic lakes in the high-plateau play as a new local geomorphological base level unaffected by the evolution of the lower regions, until either the lake becomes overfilled by sediment, overtopped by water, tectonically undammed, or captured by a neighbour drainage basin [49]. The rivers in Fig 8 show this competition very clearly: the central part of the plateau drains first towards the west (10 Myr, Fig 3), then becomes endorheic (20 Myr), then is captured by a NE river, and finally is captured by the Tarim basin (40 Myr) when this basin becomes significantly lower than the surrounding topography. For example, the flat region at ~3400 m elevation in Fig 8b is partially inherited from the above mentioned endorheic stage in the plateau (also visible in Fig 8a), and has been partially maintained by the ongoing uplift at the sector between 500 and 1000 km (distance along the river).

The use of a strong rheological stratification as in our reference model setup MS0 (e.g., [50]) promotes the localization of thickening near the indenter. In contrast, the use of a weaker lithospheric rheology in the setup MS1 amplifies the propagation of deformation towards the far continent (Fig 6) because the gravitational forces become more important relative to the tectonic ones. This is consistent with previous results from thin-sheet modelling [46]. For the purpose of this paper, what matters is that our reference model predicts a propagation of deformation and surface uplift towards the north of the *Tarim* block that roughly matches previous reports for the Tien-Shan range [51].

Now that the model’s response in terms of tectonic deformation and surface erosion/transport is calibrated by comparing with a real scenario, we can assess whether the climatic/surface processes exert any influence on the rates of crustal thickening. To do this we compare the reference model with an identical setting in which surface sediment transport is inhibited. Fig 7 shows the profiles of various parameters of the model resulting for both models (with and without erosion/sedimentation), for two cross sections than cross the collision zone approximately perpendicular to the structures. One of the sections goes NW-SE across a corner of the indenter (the western syntax) and the other one is oriented N-S to cut through the rigid *Tarim* block. Both sections show the orographic precipitation *P* and the erosion localizing in the southern side of the maximum topography, but because the western indenter corner faces the wind’s NW direction, the maximum *P* values are obtained in that area. In the real world, the highest precipitation rates are in fact measured near the eastern Himalayan syntax (, due to seasonal monsoon variations that are beyond the scope of this study. Sediment thickness in the foreland basin south of the plateau (equivalent to the Indus-Ganges basin in the Asian analogue) is also maximized in the corner area (see also Fig 5), in this case because the accommodation space is larger there due to the 3D flexural effect in response to the curved load of the plateau (rather than due to increased sediment supply; see also [52]). The way erosion modifies topography along both sections is quite intuitive. The interesting point is how this mass removal from the surface enhances the subsequent rates of thickening due to a reduction in lithostatic pressure at depth. The possible role of erosion in localizing tectonic deformation is a subject of intense research and debate since two decades ago (e.g., [53]). The SE-NW profile (Fig 7) shows a 50% increase in thickening rate (vertical strain rate) relative to the no-erosion model MS2, at *t* = 50 Myr. In other words, the high erosion rates localized at the southern side of the plateau delay the forward propagation of plate thickening towards the northern (undeformed) parts of the continent. The exhumation of 16 km of rock from the highest elevations leads to a prolongation of thickening in those areas, as if the thin sheet deformation tends to attain a similar topographic distribution as in the no-erosion model (MS2).

Interestingly, this effect is less noticeable in the N-S section. A systematic parameterisation has shown that this is caused by the presence of the *Tarim* rigid block, which constrains the patterns of deformation more efficiently than surface processes do. This explains why the overall topographic configuration does not change dramatically for the different climatic settings (MS0, MS3, MS4 in Fig 5) and why the effects of orographic precipitation on tectonics are less apparent in topography than in the erosion rates or in the thickening rates. In fact the main topographic differences between the models in Fig 5 are visible in the intramountain basin formed on the rigid intracontinental block (*Tarim*), but this is more related to the sensitivity of this lake environment to the local drainage conditions (sediment supply; endorheic or exorheic drainage) imposed by the wind direction than to the relative small effects of erosion on tectonic deformation.

## Discussion

The reference model reproduces some trends of the topographic evolution of the Tibetan Plateau, having into account the limitations of both the model and the available observations. It is of little surprise that the long-wavelength final topography predicted in the model is too low compared to the Tibetan Plateau, since it has been previously shown that mechanisms unaccounted here such as lithospheric mantle removal, are required to explain part of that elevation [19], [26]. Although we do not attempt a reproduction of the Indian-Asian collision, the final extension of the thickened crust is comparable to that of Asia related to the India indentation [54]. Since also the first-order precipitation rates and sedimentary thickness are reproduced [54], and because the evolution is linked to independently-tested physical-mathematical models, we give some credibility to the process interactions found.

The timing of both rock and surface uplift in the Tibet is a matter of intense discussion with paleodrainage, paleoelevation, and paleoclimatic evidence being interpreted as both favouring northeastward growth of the plateau [55], [56] or a rather synchronic rise of the topography of the Tibetan Plateau [51]. The model is consistent with an overall northward propagation of crustal thickening and uplift rates, although this propagation is far from linear and the plateau grows simultaneously in the vertical and horizontal directions (see for example the evolution of the elevation profile A-B’ in Fig 7). However, this style of tectonic deformation is mostly dependent on the rheological model adopted (activation energy, etc), which, as in previous geodynamic models, is still poorly constrained from rock mechanics studies. It is however interesting to highlight how this deformation interacts with the surface processes: Because neither rock uplift nor the river network erosion and transport take place at constant rates, the model does not reach anything comparable to a topographic steady state in which uplift is approximately compensated by erosion rates (compare the evolution of vertical strain rates and erosion in Fig 6; see also accompanying multimedia material). This result suggests that the topographic steady state at the orogenic scale envisaged from previous models [6] may not hold at continental scales when the tectonic deformation propagates far into the continental interiors.

The climate continentalization of the Asian interior has been previously linked both to the surface uplift of the Tibetan Plateau and to global cooling. The lack of consensus on the age of aridification prevents the elucidation of the relative quantitative importance of these factors [57], [58], but the results are consistent with recent dune dating that suggest that the Tarim desertic weather is at least Miocene in age [58]. This aridification of the continent interior facilitates the incorporation of basins and the lateral growth of the plateau in the way proposed by [59], [60] and modelled by [40].

The model explicitly accounts for the interplay between lithospheric and surface processes in both directions: first, surface processes determine the way mass is redistributed in space and time, thus modifying the isostatic vertical motions and the subsequent tectonic deformation that accommodates plate motions. Secondly, the crustal and lithospheric tectonics control and organize the non-linear nature of drainage networks, and the localization of precipitation in response to topographic growth. In zones deformed by folding and thrusting, the local drainage evolution is controlled by the kinematics of such tectonic deformation. Other mechanisms such as lithospheric flexure can become relevant in determining the drainage network in less deformed areas such as at the external margin of foreland basins [31], [61] or during the post-tectonic erosional rebound of an escarpment [62]. In the continental scale, though, the presence of domains or block with distinctive strength seems to be determinant of the drainage and topographic evolution.

**Fig 9:**
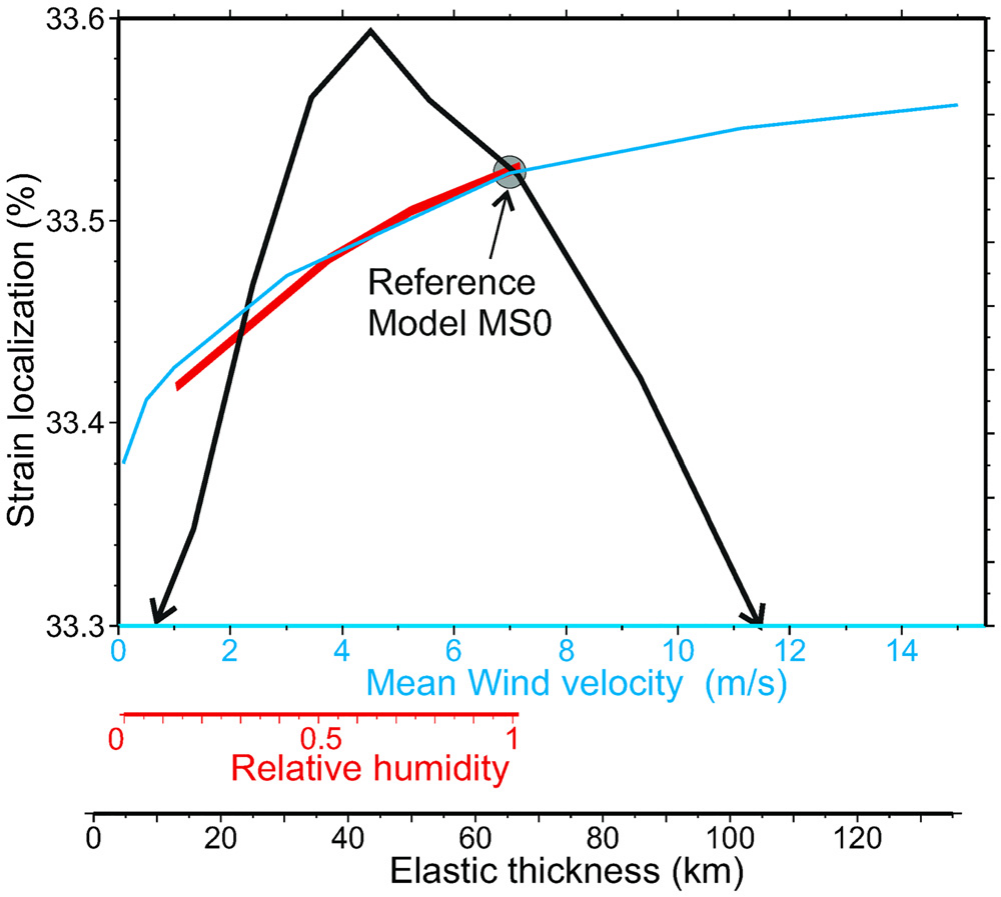
Parameterization. Dependence of the average tectonic strain localization (%) on orographic effects (incoming air humidity *R*_*H*_ and mean wind velocity *u*_*w*_) and on the lithospheric elastic thickness. Strain localization is measured as the standard deviation of the accumulated vertical strain (dimensionless). Note that although the changes in this averaged are smaller than 1%, strain rates can locally be enhanced by 50% in areas of rapid erosion (Fig 7a). The reference setup is located with a circle.

The results show that structural rheological contrasts such as the pre-existence of stronger lithospheric domains in the plate are more relevant in determining the localization of deformation, sedimentary basins, and topographic highs and lows, in front of climatic/erosional heterogeneities (note the similar overall topographic patterns predicted in all models in Fig 5). Orographically focalized erosion can locally modify by up to 50% the rates at which the lithospheric thickening takes place in the weaker regions. This is best shown by the differences induced in thickening rate in the AB’ section in Fig 7, but we have systematically estimated this effect by defining a measure of strain localization as the standard deviation of the accumulated vertical strain, *ε*_*33*,_ at *t* = 50 Myr relative to its average value. Fig 9 shows how this strain localization changes with the mean wind velocity, the relative humidity and elastic thickness. The speed and humidity of the incoming air induce proportional changes in precipitation and erosion, and higher differences in strain localization. But note that these variations amount to less than 1% relative to the average strain in the entire model, and that even an unsuspected parameter as the elastic thickness of the flexural plate can have a larger effect.

The atmospheric enhancement of tectonic crustal thickening is more efficient at the corners of the indenter. While deformation concentrates preferentially along these syntaxes regardless of the precipitation they concentrate, the amount of strain they accommodate can be enhanced by localized orographic precipitation by up to 50%, relative to a model with laterally-constant precipitation (Fig 7a). It has been long debated that the extreme erosion rates observed in the syntaxes of the Himalayas (locally above 6 mm/yr) may be responsible for rapid tectonic rock uplift causing the so-called tectonic aneurysms [63], [64]. While our results show an enhancement of up to 50% in rock uplift rates by localized orographic precipitation, it must be kept in mind that the tectonic model is limited by a vertically-averaged rheology and that neither plate subduction [65] nor mantle delamination are here accounted for. Previous studies have shown that erosion and deposition can have a larger influence on the crustal/lithospheric structure and on topography at the scale of an orogen or a plate subduction [66], [2], [10]. An erosion-triggered lateral tectonic flow was proposed by [67] to explain the rapid lateral exhumation of the Greater Himalayan Sequence, implying a mechanical decoupling between mantle and crust. Our model implicitly assumes crust-mantle coupling, in agreement with previous mechanical modeling constrained by earthquake focal mechanisms [68]. So our results suggest that even for a coupled lithosphere are the effects of erosion on tectonic deformation expected to be locally significant in areas of localized orographic precipitation while they become negligible at the continental scale.

**Fig 10:**
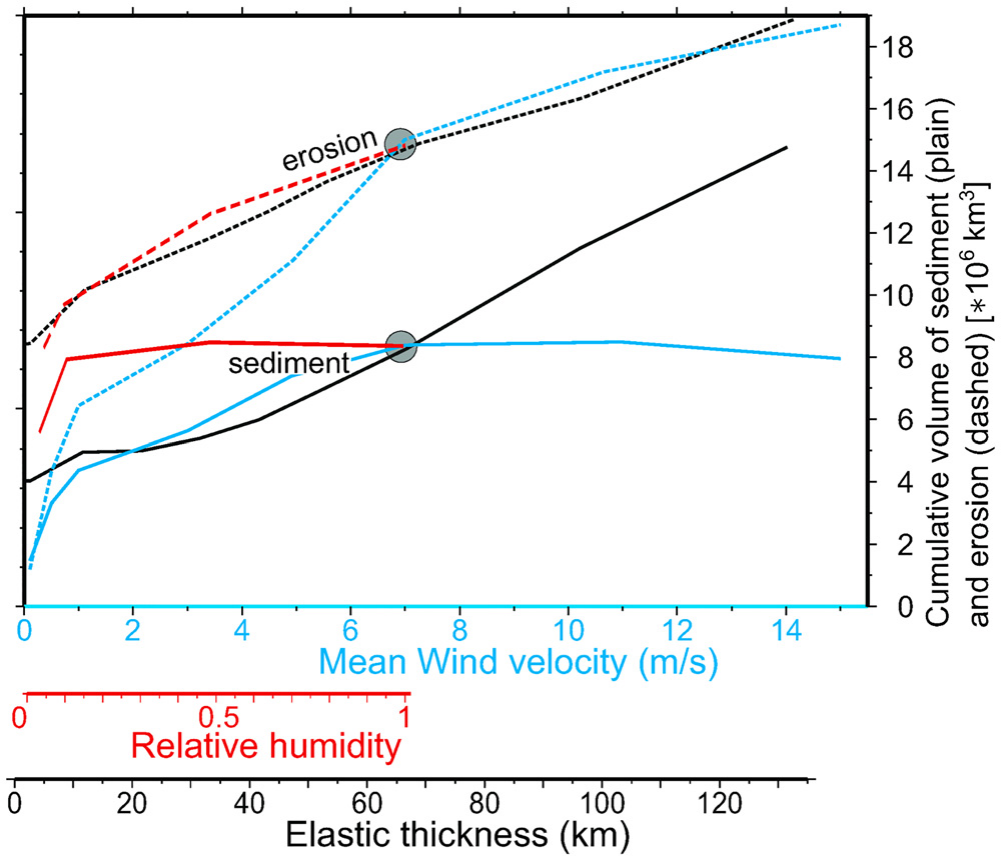
Parameterization. Sediment volume vs. elastic thickness *Te* and precipitation *P*. The reference setup is located with circles.

The effects of the climate parameters of the model become more significant in terms of surface mass redistribution. Fig 10 shows a systematic parameterization of the total amount of eroded rock and sediment accumulation at t = 50 Myr as a function of the speed *u*_*w*_ and humidity *RH* of the incoming wind and the lithospheric elastic thickness *T*_*e*_. All three parameters produce a comparable enhancement of erosion and sediment storage within the model domain. It is worth noting that this positive correlation does not stand for large values of *u*_*w*_ and *RH*, which inhibit endorheism. Humid conditions cause the Tarim-like basin, for example, to overflow and drain towards the west, excavating a gorge along the outlet similar to what happens in model MS4 (Fig 5) and reducing the accommodation space available for sediment. This provides an alternative mechanism for the documented large-scale river captures in the east-Tibetan drainage [69], previously suggested to be a record of a late uplift event [26] perhaps related to subcrustal processes and dated as Miocene or later. The transition from endorheic to exorheic conditions may have been also triggered by a climatic change to more humid conditions.

## Conclusions

**Fig 11:**
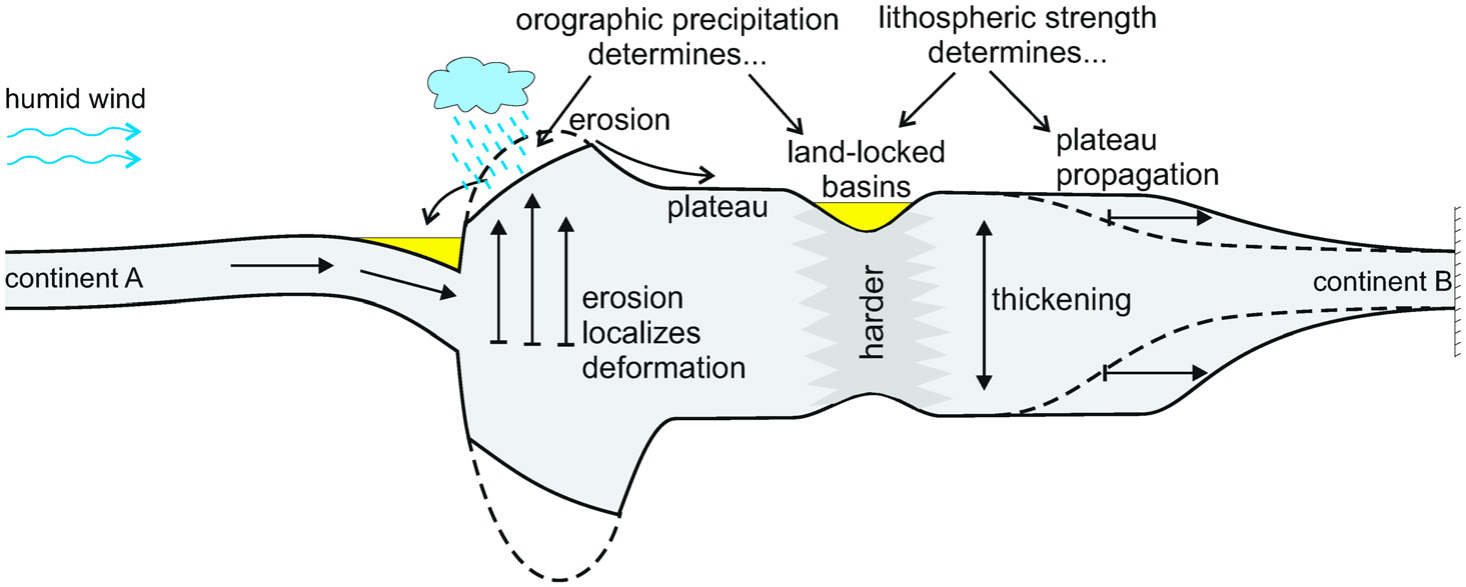
Interpretative cartoon of the interplay between surface and lithospheric processes.

We show a first attempt to describe the evolution of topography at continental scales accounting for a process spectrum ranging from continental collision to orographic precipitation. A detailed set of mathematical formulations for these processes and the associated computer code to solve them are provided with this paper. Under the approximations adopted for this work and subject to the aforementioned limitations, the following conclusions (summarized in Fig 11) can be made:

1. At the continental scale, the main controls on the evolution of topography are the motion of tectonic plates and the inherited distribution of strength in the lithosphere. Surface processes reduce large-scale relief according to the climatic conditions, and feedbacks exist between topographic growth, orographic precipitation, and surface sediment transport, but these seem insufficient to substantially change the large-scale emplacement of focalized deformation.
2. Erosion has a limited but significant effect on the distribution of crustal and lithospheric deformation at continental scales. Localized precipitation in the up-wind side of an orogeny leads to abrupt lateral changes in erosion rates. The removal of tens of km of rock from the surface in turn modifies the lithostatic pressure distribution at deep crustal and lithospheric levels, modifying the accommodation of further tectonic shortening. In a Tibet-scaled scenario, the model estimates a <50% increase in deformation rates at the southern edge of the plateau (Himalayas) due to this effect.
3. In presence of inherited tectonic structures of moderate strength contrast, these dominate over climatic/erosional effects in shaping tectonic deformation, and the erosion/sedimentation effects become hardly detectable. For example: In our synthetic scenario, the deformation rates along a N-S transect from the Himalayas to the Tarim basin shows variations of <15% when changing between extreme-case climatic scenarios.
4. The aridification of climate resulting from the growth of the orographic rain shadow during continental collision enhances intracontinental sediment trapping by promoting endorheism (internal-drainage) in the plateau and the lee side of the orogen.
5. The evolution of the drainage patterns responsible for surface rock redistribution and the location of sedimentary sinks can be significantly modified by climatic factors such as the evaporation regime and by orographic precipitation patterns. Enhanced rain in the upwind side of the continent and enhanced evaporation/precipitation ratios in the lee side determine the sediment and water overflow of sedimentary basins, and their possible colmatation and erosion. Large-scale drainage changes (river piracy) may have been favored by the presence of land-locked plateau basins that are captured as they grow in elevation and become eventually overfilled by sediment or water. This adds to the possible role of an abrupt tectonic uplift at the eastern Tibetan Plateau, proposed by [69]) and [26]). Surface drainage changes, together with the non-linear propagation of tectonic deformation into the continent, make unlikely the development of a topographic steady state at the continental scale.

## Supporting Information

S1 File Video of the evolution of reference model MS0. The left panel shows the evolution of topography and drainage, with areas where precipitation exceeds 400 mm/yr shaded in red. The right panel shows the erosion rate (shade) and the thickening rate (contours) in response to tectonic convergence and continental collision.
(MOV)
